# Long Single-Stranded DNA with Site-Specific Modifications to Enable Single-Base-Resolution Epigenetics

**DOI:** 10.1002/anie.202513078

**Published:** 2025-11-19

**Authors:** Chenyou Zhu, Yin Xu, Ziyang Hao, Yuan Zhuang, Rui Xu, Yufan Pan, Jiezhong Shi, Yujie Li, Yifan Jiang, Tianran Wang, Yuhe R. Yang, Huajie Liu, Dali Han, Yuanchen Dong, Chuan He, Dongsheng Liu

**Affiliations:** [a] Engineering Research Center of Advanced Rare Earth Materials (Ministry of Education), Department of Chemistry, Tsinghua University, Beijing, 100084, China; [b] China National Center for Bioinformation, Beijing Institute of Genomics, Chinese Academy of Sciences, Beijing, 100101, China; [c] School of Pharmaceutical Sciences, Capital Medical University, Beijing, 100069, PR China; [d] CAS Key Laboratory for Biomedical Effects of Nanomaterials & Nanosafety, CAS Key Laboratory of Nanosystem and Hierarchical Fabrication, CAS Center for Excellence in Nanoscience, National Center for Nanoscience and Technology, Beijing, PR China; [e] School of Chemical Science and Engineering, Shanghai Research Institute for Intelligent Autonomous Systems, Key Laboratory of Advanced Civil Engineering Materials of Ministry of Education, Tongji University, Shanghai 200092, China; [f] CAS Key Laboratory of Colloid, Interface and Chemical Thermodynamics, Institute of Chemistry, Chinese Academy of Sciences, No. 2 Zhongguancun Beiyijie, Beijing, 100190, PR China; [g] Department of Chemistry, Department of Biochemistry and Molecular Biology, Institute for Biophysical Dynamics, The University of Chicago, Chicago, IL 60637, USA; [h] Howard Hughes Medical Institute, The University of Chicago, Chicago, IL 60637, USA; [i] Department of Applied Biology and Chemical Technology, The Hong Kong Polytechnic University, Hong Hum, Kowloon, Hong Kong, China; [j] PolyU Shenzhen Research Institute, The Hong Kong Polytechnic University, Shenzhen, 518057, P.R.China

**Keywords:** Long single-stranded DNA, Site-specific modifications, Epigenetics, Single-base resolution, 5-methyl cytosine

## Abstract

We reported a method for synthesizing site-specifically modified long single-stranded DNA (ssDNA) with high purity and fidelity. With the successful synthesis of long ssDNA carrying different 5-methylcytosine (5mC) patterns, single-base-resolution analysis of 5mC epigenetic dynamics at specific genomic loci were enabled in mammalian cells. The results revealed that the density of 5mC sites synergistically correlated with their maintenance. This study opens new avenues for precise epigenetic manipulation and therapeutic applications.

## Graphical Abstract

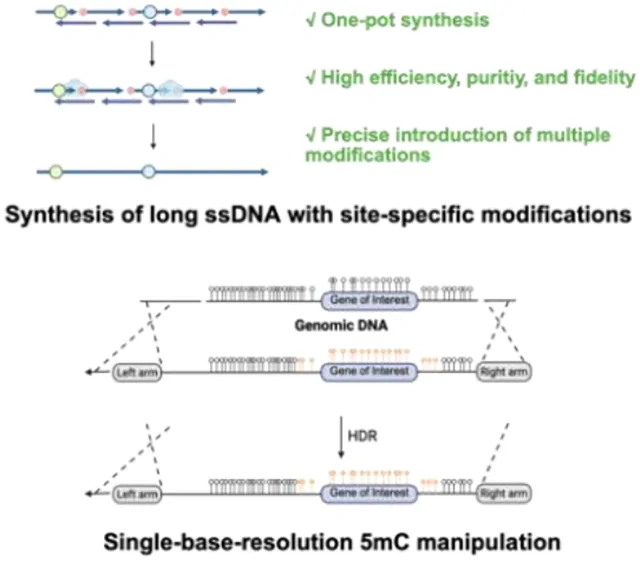

We report a robust one-pot synthesis of long single-stranded DNA (ssDNA) with precise site-specific modifications. When applied to 5-methylcytosine (5mC) patterns and CRISPR/HDR, this method enables single-base resolution epigenetic manipulation and uncovers synergistic density effects in methylation maintenance.

## Introduction

Chemical synthesis of long single-stranded DNA hold significant potential for numerous applications ranging from gene therapy, synthetic biology, epigenetic control, and information storage^[1–5]^. There are currently limited methods to prepare long stretches of DNA largely because of challenges to purify final products from chemical synthesis. Enzymatic methods may not be able to handle long DNAs bearing repeat sequences or unbalanced sequence contents. Furthermore, enzymatic approaches for modifying long DNAs are constrained by the context-dependent specificity of enzymes, which limits the ability to introduce modifications at arbitrary positions along the DNA sequence. Currently, methods capable of generating long DNA constructs bearing precisely controlled modifications at user-defined sites are scarce 5-methylcytosine (5mC) is a pivotal epigenetic mark that plays a critical role in regulating various physiological processes, such as development, aging, and cancer^[6–10]^. Precisely interpreting the causal relationship between functions of 5mC and its spatial and temporal deposition is critical to the understanding of gene expression regulation. The emerging high-throughput sequencing technologies have enabled genome-wide mapping of 5mC at single-base resolution; a comparable method to implant 5mC with equivalent precision remains unavailable. Such a method would be important to reveal the exact correlations between 5mC patterns and gene regulation^[11–13]^. Over the past decades, numerous efforts have been made to manipulate 5mC modifications in living systems. Pharmacological or genetic perturbations were adopted to induce genome-wide changes in 5mC, often led to the pleiotropic results^[14]^. More recently, advances in gene editing technologies have made it possible for targeted methylation through the fusion of effector proteins with addressable proteins such as zinc finger proteins (ZFPs), transcriptional activator-like effectors (TALEs), and CRISPR/Cas proteins^[15–18]^. Among these, CRISPR-Cas systems have emerged as a powerful tool for locus-specific manipulation of 5mC enabling methylation typically within a 25–35 bp window flanking their targeted sites,^[19]^. However, limitations such as insufficient editing resolution, substantial off-target activity, and interference effect due to competition with transcription factors hindered the comprehensive manipulation of 5mC^[20,21]^.

Homology-directed repair (HDR) offers a direct way to introduce distal and global 5mC methylation into genomic loci^[22]^. To achieve optimal HDR efficiency, the donor DNA spanning several hundred nucleotides, comprising a central modification region harboring site-specific methylated CpG sites and two flanked homologous arms. However, the chemical synthesis of modified ssDNA exceeding 300 nt remains a significant challenge due to constraints in yield and purification^[23]^. This length falls well below the requirements for HDR mediated long-range 5mC editing. As the length of ssDNA increases, conventional purification methods, such as high-performance liquid chromatography (HPLC) and polyacrylamide gel electrophoresis (PAGE), fail to distinguish the desired ssDNA from impurities of comparable length, such as truncated by-products differing by minimal nucleotides. To address this resolution limitations, the traditional stepwise coupling of individual nucleotide monomer during chemical synthesis can be supplanted by the modular ligation of oligonucleotide segments. Nevertheless, the sequential ligation of multiple fragments is labor-intensive and inefficient, highlighting the necessity for the development of one-pot synthesis approach.

Herein, we report a facile and reliable one-pot method for synthesizing long ssDNA. This approach employs the ligation of synthetically modified DNA fragments through a kinetically-locked assembly, resulting in a long stretch of DNA products (> 700 nt). We showcase a key application of this approach by preparing long ssDNA with programmable and site-specific modifications. Various modifications such as fluorophores, phosphorothioate, and 5mC were precisely introduced at specific sites of long ssDNA. By integrating long ssDNA bearing pre-programmed 5mC modifications via homology-directed repair (HDR), our method enables precise incorporation of 5mC at arbitrary sites, allowing for site-specific incorporation of 5mC sites across extensive sequences for epigenetic characterizations in live cells for the first time. Furthermore, by manipulating the methylation landscape of the GSTP1 promoter in HEK293T cells at single-base resolution, we discovered that the density of these 5mC sites correlated with their epigenetic maintenance. With higher 5mC density on the ssDNA donor, the greater maintenance of 5mC in the native genome will be observed.

## Results and Discussion

### System Design

**Scheme 1.**
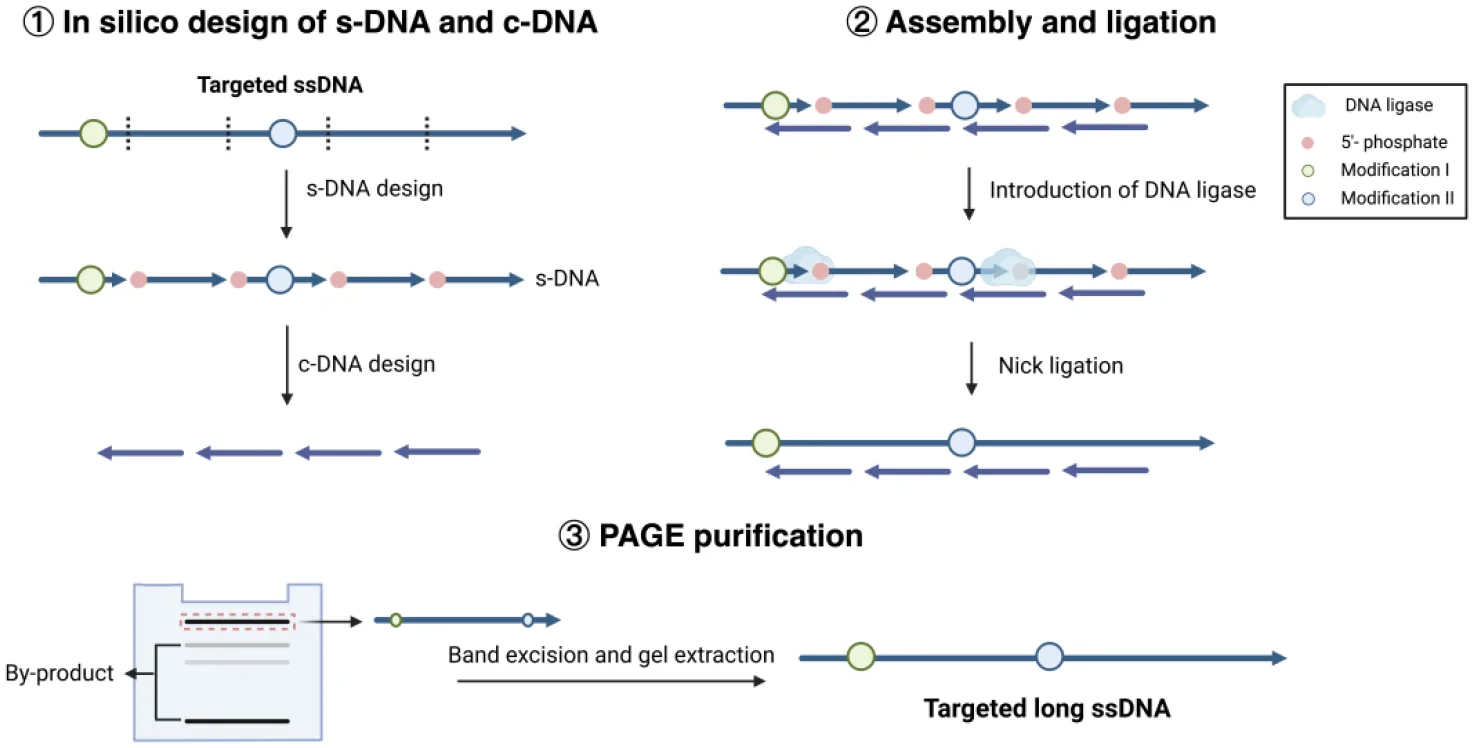
Schematic illustration of synthesis of long ssDNA with site-specific modifications. The synthesis could be divided into three steps. Firstly, targeted long ssDNA was cut into multiple s-DNAs with pre-disposed modifications and 5ʹ phosphate. Then, corresponding c-DNAs which hybridize with c-DNA were in silico designed. Secondly, s-DNAs and c-DNAs were synthesized by an oligo synthesizer and assembled. T4 DNA ligase was introduced to obtain the targeted long ssDNA. Finally, denaturing PAGE was used for purifying the targeted long ssDNA from other by-products.

Our method is based on the ligation of kinetically-locked assembly of multiple oligos (Scheme 1). To synthesize a targeted long ssDNA, we started with designing the segmented DNA (s-DNA) by dividing the target sequence into distinct fragments. The complementary DNA (named c-DNA) was derived to hybridize with two adjacent s-DNA in an overlap fashion. In our design, only the s-DNA was phosphorylated, ensuring the subsequent enzymatic ligation of targeted long ssDNA assembled with multiple short c-DNAs, which can be facilely denatured to produce the target ssDNA. The s-DNA and c-DNA will from fully nicked duplexes without any gap. Moreover, to enhance the resolution between the targeted product and by-products, the length of s-DNA was maintained above 20 nt. Then, equivalent amount of s-DNA and c-DNA would be assembled into kinetically-interlocked linear duplex with high stability and efficiency. Finally, the aligned nicks were subsequently ligated by DNA ligase (e.g., T4 DNA ligase) to produce the full-length ssDNA. By pre-disposing targeted modifications into the short oligos units, a series of long ssDNA with precisely positioned modifications were successfully prepared.

To optimize the synthesis efficiency of long ssDNA, the sequence of the s-DNAs and c-DNAs must be carefully designed to form a stable linear duplex with high efficiency. It is known that the assembly process is affected by the binding affinity of s-DNA and c-DNA, which can be assessed through the melting temperature (*T*_m_). Therefore, Tm of each overlap between s-DNA and c-DNA should be as close as possible to minimize unexpected binding preferences, a principle consistent with the recently reported KIMU model^[24]^. To investigate the influence of Tm on assembly efficiency, we have applied a typical system to construct a random 200 bp duplex, testing three different segmenting designs with Tm values of 25 °C, 35 °C and 45 °C (Detailed sequences are provided in Table S1). After annealing of the equivalent mixed s-DNA and c-DNA from 95 °C to room temperature, the resulting assemblies were characterized by native PAGE (Figure S1). At the designed Tm of 45 °C, a new single band with slower migration was observed, demonstrating the successful formation of the target assembly. In contrast, at Tm value of 25 °C and 35 °C, only bands corresponding to individual s-DNA and c-DNA were detected, suggesting unsuccessful assembly under such conditions. These results have demonstrated the critical role of sequence design in modulating the dynamic assembly process through Tm optimization, establishing a foundation for subsequent ligation steps.

### Synthesis and Purification of Long ssDNA

**Figure 1.**
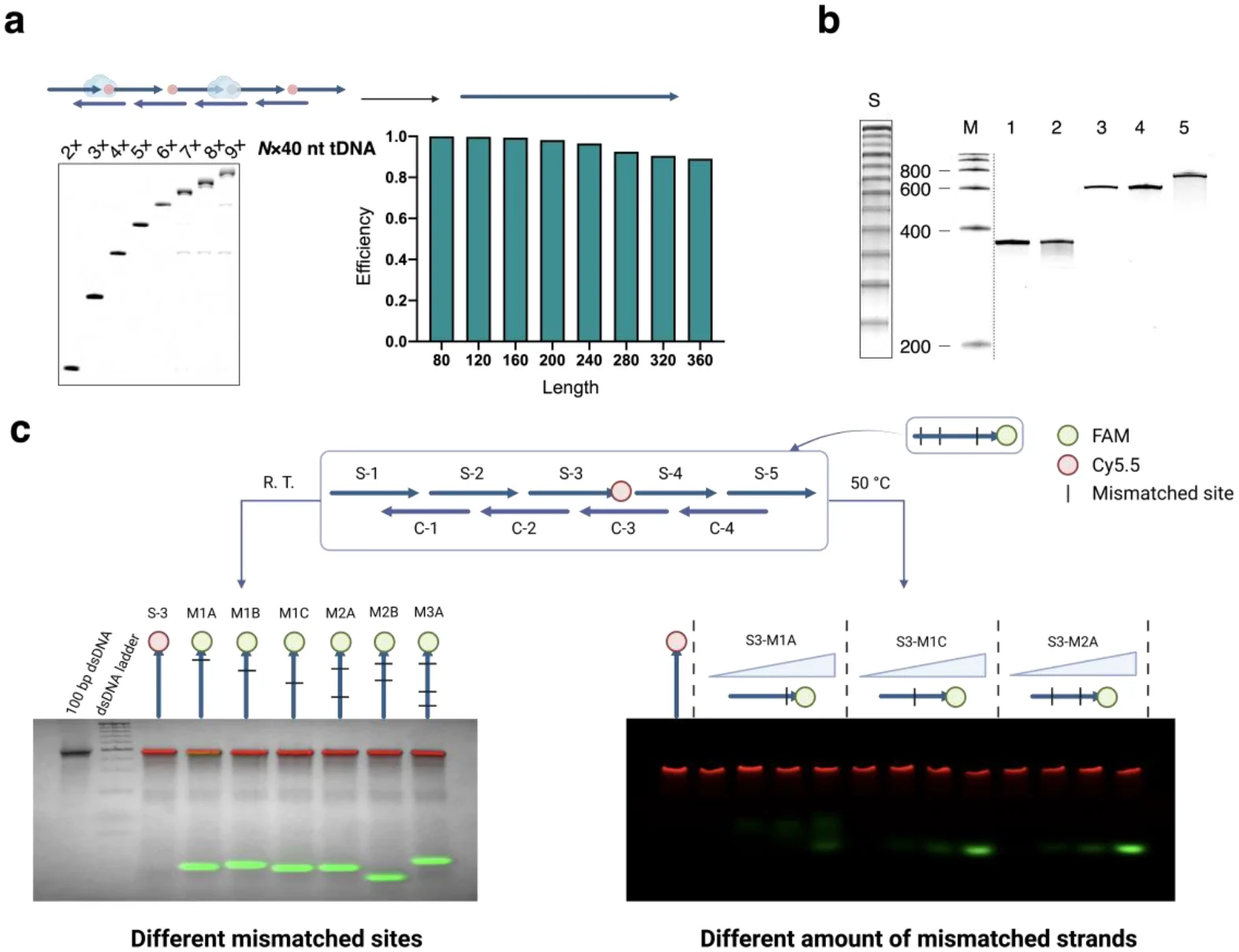
Synthesis of long ssDNA and self-proofing ability of the strategy. (a) One-pot synthesis of 80 nt to 360 nt ssDNA. Left, 6% denaturing PAGE characterization of synthesis mixture under FAM channel. Right, efficiency of synthesizing 80 nt to 360 nt ssDNA. The synthesis efficiency was determined by dividing the fluorescent intensity of the target full-length ssDNA band by the sum of all visible band intensities. (b) One-pot synthesis of multiple long ssDNA. Left, 6% denaturing PAGE characterization of synthesis mixture of 720 nt ssDNA. Right, 6% denaturing PAGE characterization of purified ssDNA with different sequence. Lanes in gel: M, ssDNA ladder; 1, 360nt-1 ssDNA; 2, 360nt-2 ssDNA; 3, 720nt-1 ssDNA; 4, 720nt-2 ssDNA; 5, 820nt ssDNA. (c) inherent error-proofing assembly of the methods. 10% native PAGE (1×TBE) was used to characterize the error-proofing ability of the methods at different temperature. Mismatched sites in each DNA strand is denoted as a horizontal line above each lane. FAM (green) for mismatched strands and Cy5.5 (red) for correctly matched strands. Left, overlay of bright field (stained with Stains All), FAM and Cy5.5 channel, with a 20 bp dsDNA ladder included to provide size reference that confirms the 100 bp size of the assembled products. Right, overlay of FAM and Cy5.5 channel.

To synthesize an arbitrary ssDNA, the s-DNA and c-DNA were firstly designed as discussed above (Sequence can be found in Table S1) and synthesized by oligo synthesizer. In a representative experiment, these s-DNAs and c-DNAs were mixed and assembled by annealing from 95 °C to room temperature, and then the T4 DNA ligase was subsequently introduced to ligate 5ʹ-phosphate and 3ʹ-hydroxyl between s-DNA. We employed denaturing PAGE to characterize the ligation complex, revealing a topmost band corresponded to our targeted long ssDNA with additional bands below indicating incompletely ligated intermediates (Figure 1b). We analyzed the band intensities corresponding to targeted ssDNA and incompletely ligated products, confirming that the efficiency is as high as 98% for the 100 nt synthesis (Figure S2). Notably, our strategy can accommodate stoichiometric and concentration deviation of DNA segments, providing significant redundancy on the assembly process (Figure S3–4). Additionally, ssDNA ranging from 120 nt to 200 nt were also synthesized with efficiency from 97.6% to 84.3% (Figure S5). Our study also revealed that a 10 min ligation was sufficient to attain a peak efficiency of 90% (Figure S6). To synthesize longer ssDNA while maintaining high assembly efficiency, the melting temperature (*T*_m_) of c-DNA and s-DNA was optimized. In principle, to ensure the sufficient resolution between target ssDNA and by-products and the formation of stable s-DNAs/c-DNAs assembly, the length of s-DNAs should be longer than 30 nt. Furthermore, the length of s-DNAs and c-DNAs should accommodate the HPLC purification process to be less than 30 nt. Target ssDNA was purified from synthesis mixtures using denaturing PAGE. Using two 360 nt ssDNA constructs as examples, the overall yields after assembly, ligation, and gel purification were 23.8% and 18.0%, respectively (Figure S7a). The yields of synthesized DNA have also been investigated systematically, which varied from 62.4% to 5.1% for lengths between 100–720 nt. As shown in Figure 1a–b, we successfully prepared a series of ssDNA from 80 nt to 720 nt with high efficiency. ssDNA longer than 1000 nt can also be synthesized in a one-pot reaction (Figure S7b). Denaturing PAGE analysis confirmed that the topmost band, corresponding to the desired long ssDNA, was distinctly separated from by-products, facilitating the efficient purification. This clear resolution can be attributed to substantial length differences between the targeted ssDNA and incompletely ligated intermediates. By employing denaturing PAGE for gel purification, we successfully obtained long ssDNA of various lengths with high purity (Figure 1b and S7). Overall, these results demonstrate that we have successfully developed a facile, reliable and efficient strategy for the synthesis and purification of long ssDNA.

We further evaluated the capability of our method to synthesize ssDNAs with extreme base compositions and repetitive sequences. Two 300 nt ssDNAs were designed with 80% G/C and 80% A/T content, respectively (sequences can be found in Table S4). For these sequences with unbalanced base compositions, the s-DNA length requires careful optimization. Specifically, we employed 30 nt s-DNAs for high G/C content synthesis to reduce the probability of intramolecular assembly, and 50 nt s-DNAs for high A/T content synthesis to ensure sufficient duplex stability. Both sequences were successfully synthesized with high efficiency (Figure S8a), demonstrating the robustness of our method across diverse sequence compositions. We also examined the synthesis of repetitive sequences, which pose significant challenges for enzymatic amplification methods. A 192 nt ssDNA containing eight tandem repeats of a 24 nt sequence was constructed using a single 24 nt s-DNA unit (Figure S8b, sequence can be found in Table S4). Following assembly and ligation, we observed efficient formation of products with varying numbers of repetitive units, indicating high assembly and ligation fidelity (Figure S8c). The resulting ssDNA with different repeat numbers were well-resolved by denaturing PAGE, enabling precise purification of the desired 8-repeat product. These results demonstrated that our method is able to synthesize long ssDNA with extreme sequence context.

### Self-Proofing Ability

The fidelity and accuracy of the long ssDNA are crucial for various applications, such as gene synthesis and gene editing. According to the mechanism of our strategy, errors in targeted long ssDNA originated from inaccuracies during the solid-phase synthesis of s-DNA fragments. Apart from using HPLC purification to ensure the fidelity of s-DNAs, the stability difference between fully complementary and mismatched DNA segments during the assembly process confers our strategy with intrinsic error-proofing ability. To validate this, we evaluated the ability of our method to exclude the errors in s-DNA by assessing the proportion of mismatched s-DNA with varying numbers and positions of mismatches within the targeted assembly prior to enzymatic ligation (Figure 1c). In a representative 9-strand assembly system, the 3ʹ end of fully complementary S-3 was labeled with Cy5.5 (red band), while the 3ʹ end of oligos with different numbers and positions of mismatched bases was labeled with FAM (green band). Mismatched oligos were added equivalently with other s-DNA and c-DNA. The assembly was characterized by thenative PAGE at different temperatures and visualized under different fluorescent channels. When an equivalent amount of mismatched strands was mixed with S-3 and assembled, most mismatched strands (green band) were excluded from the targeted assembly mixture, demonstrating the robust error-proofing capacity (Figure 1c, left image). This error-proofing ability was further enhanced at an assembly temperature of 50 °C. When different amounts of three selected mismatched strands were included in the assembly and characterized by native PAGE at 50 °C, no incorporation of mismatched strands into the targeted assembly was observed, indicating improved error-proofing ability at higher temperature (Figure 1c, right image). Beyond assembly characterization, the fidelity of synthesized long ssDNA was directly confirmed by Sanger sequencing of the final product following amplification by polymerase chain reaction (PCR) and cloning into a vector. As a representative example, we assessed the fidelity of a synthesized 720 nt ssDNA by Sanger sequencing 40 independent subclones. The average error rate per base was determined to be 0.038%. (Figure S9a). Using the same method, we further measured the average error rates of other ssDNA from 300 nt to 1228 nt. The average error rates were calculated to be between 0.017% and 0.097%, further supporting the high fidelity of our method (Figure S9b–d). It should be noticed that only a single mismatch, insertion, or deletion was observed in the imperfect clones, and the sequencing error did not overlap between clones, suggesting that these discrepancies may stem from the inherent errors in PCR or Sanger sequencing process.

### Synthesis of Long ssDNA with Various Site-Specific Modifications

While modifications of the DNA in the genome have been recognized essentially in numerous biological processes, the site-specific modification of long ssDNA remains a significant challenge. Taking advantage of the precise modifications in short oligo synthesis, our strategy enables the facile synthesis of customized long ssDNA with modifications at single-base resolution. To showcase the versatility of this approach, we introduced fluorophores, phosphorothioate (pt) and 5mC at designated sites within long ssDNA.

**Figure 2.**
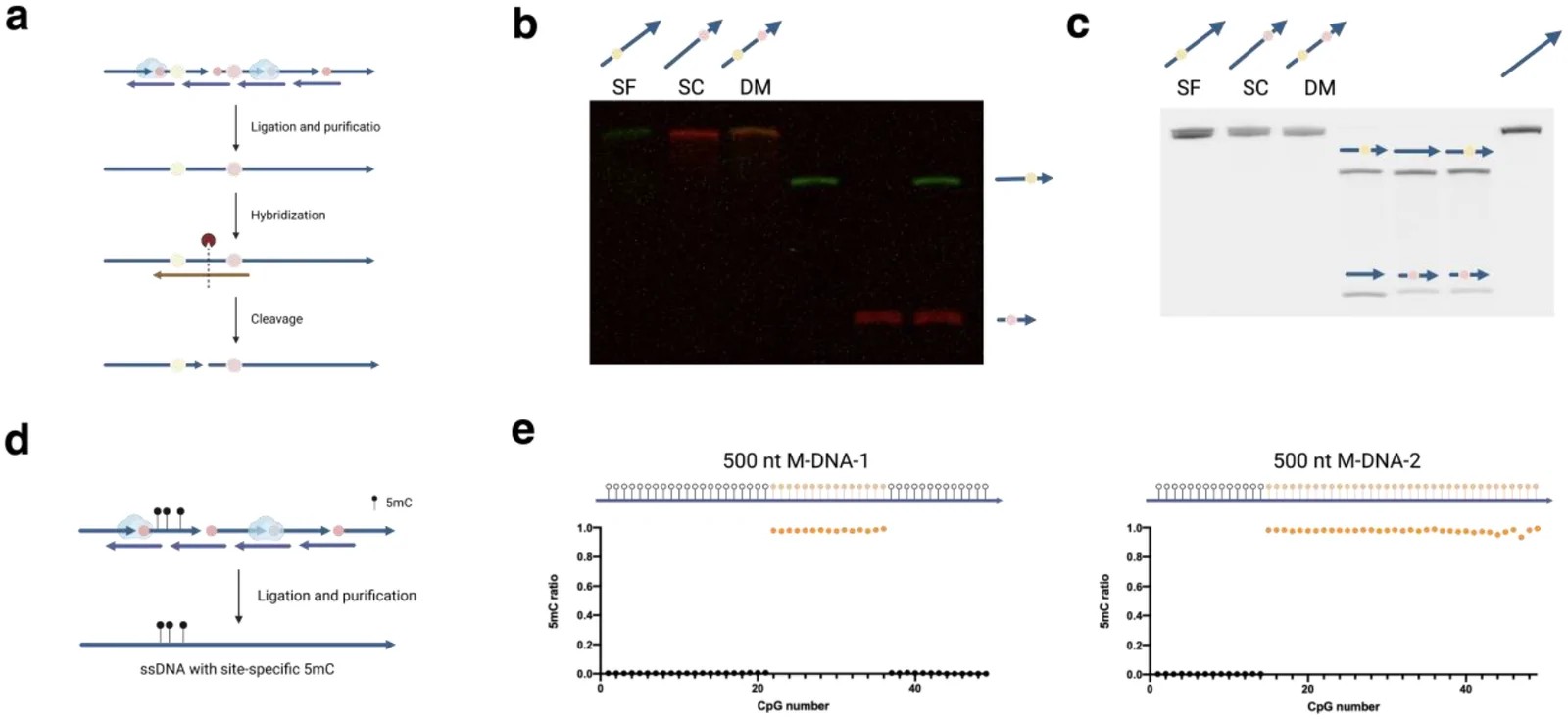
Synthesis of long ssDNA with site-specific modification. (a) Scheme of synthesis and characterization of 360 nt ssDNA with FAM and Cy5 modification at specific base. Modification on ssDNA was characterized by EheI cleavage assay and denaturing PAGE. The two modified base flanked the EheI cleavage site and two ssDNA with different length and fluorescence tag will be generated after EheI cleavage. (b) 6% denaturing PAGE analysis of cleaved product. Image is the merged channel of FAM and Cy5. Green and red band corresponds to fluorescence of FAM and Cy5, respectively. ssDNAs are synthesized from 9 s-DNAs and 8 c-DNAs. (c) 6% denaturing PAGE of synthesized 360 nt and cleaved product. Image is the channel of SYBR gold staining in Fig. 2b. (d) Scheme of synthesis of 500 nt ssDNA with site-specific 5mC modifications. ssDNAs are synthesized from 10 s-DNAs and 9 c-DNAs. (e) High-throughput sequencing results of M-DNA-1 and M-DNA-2. Purified ssDNA was treated with sodium bisulfite, PCR amplified, ligated with sequencing adapter and sequenced.

**Figure 3.**
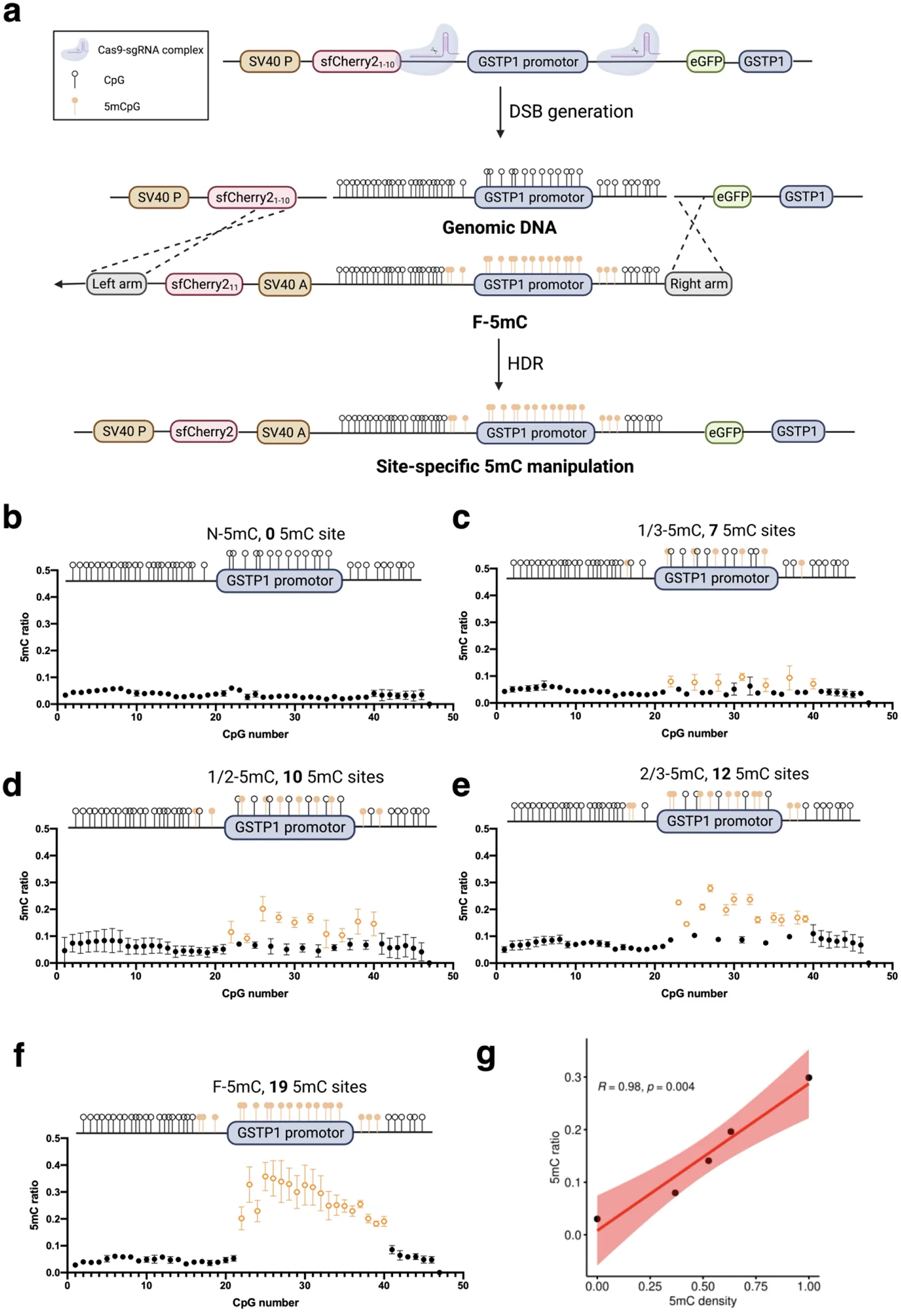
Calculation of 5mC ratio of the cellular genome after the insertion of ssDNA. (a) Scheme of integration of ssDNA donor with site-specific 5mC modifications, which uses the integration of F-5mC as an example. (b-e) 2 days after electroporation, high-throughput sequencing results of 5mC ratio of each CpG site near GSTP1 promoter in cells with integration of corresponding ssDNA. Data are mean ±s. e. m. (*n*=3 biological replicates). In each image, the methylated CpG sites in parental ssDNA were shown with orange circles, and other unmethylated CpG sites were shown with black circles. Each image contains a scheme of the detailed patterns of methylated (hollow black circle) and unmethylated CpG sites (filled orange circle) within the corresponding ssDNA donor. (g) Pearson correlation results between parental 5mC density with corresponding genomic 5mC ratio after 2 days post electroporation. In ascending order from left to right, the density of parental 5mC is as follows: N-5mC, 1/3–5mC, 1/2–5mC, 2/3–5mC, and F-5mC.

As a representative example, FAM and Cy5 were selected as target fluorophores, which could be introduced to s-DNA during its solid-phase synthesis. Specifically, we have synthesized a 360 nt ssDNA with three modification patterns: single FAM (SF) at position 247, single Cy5 (SC) at position 271, and dual modifications (DM) (Figure 2a). The modified fluorescent ssDNAs were characterized by denaturing PAGE under a fluorescence imaging system. The corresponding s-DNA has been labeled with the required fluorescence during the solid phase synthesis, and the long ssDNA with site-specific modifications can be easily prepared following our strategy. As illustrated in Figure 3a, SF and SC were visualized exclusively under their respective channels, the DM was detected under both channels, appearing as a merged yellow band (Figure 2b). To confirm the precision of these modifications, an EheI restriction site was inserted between positions 257 and 258, with the two modified base flanking the EheI cleavage site. Post-cleavage analysis of the DM sample revealed two bands corresponding to the expected segmental lengths and associated fluorophores, whereas SF and SC each displayed a single band with the respective fluorophore (Figure 2b). These results demonstrated the successful synthesis of long ssDNA with precise fluorescent modification at defined sites.

To further demonstrate the versatility of our method, phosphorothioate (pt) modification was introduced at specific sites of the long ssDNA. The distal and internal pt modifications protect long ssDNA from digestion by exonucleases and endonucleases, respectively (Figure S10). These results further indicated the robustness and adaptability of our strategy for synthesizing long ssDNA with targeted chemical modifications, thereby enabling the endowment of synthesized DNA with tailored functionalities.

Our method can also be extended to synthesize long ssDNA with site-specific methylation. As a proof of concept, we synthesized 500 nt ssDNA and introduced 5mC at selected CpG sites. Specifically, methylation was targeted at positions 22 to 36 (M-DNA-1) and 15 to 49 (M-DNA-2) among the 49 CpG sites present. The purity of the synthesized ssDNA was characterized by denaturing PAGE (Figure S11a). To verify the methylation sites on the ssDNA, the synthesized DNA was treated with sodium bisulfite and sequenced. We observed nearly 100% methylation at the targeted sites with no detectable off-target modifications across the entire strand (Figures 2d–e and Figure S11b), indicating the accurate incorporation of 5mC. These results clearly demonstrate the effectiveness and precision of our method in synthesizing long ssDNA with site-specific 5mC modifications, positioning it as a powerful tool for manipulating DNA methylation at the single-base level.

### Study of DNA Methylation Maintenance at Single Base-Resolution

Whole-genome sequencing of 5mC at base resolution has revealed a landscape of hypomethylation at CpG islands (CGIs) and hypermethylation at other CpG motifs. However, the dynamic behavior of 5mC especially the causal and correlational relationships between DNA methylation and its downstream effects, remains poorly understood. Previous studies have underscored the cis-regulation feature of 5mC at genomic loci, which indicated that the efficiency of 5mC maintenance in a given region depended on synergistic interactions among adjacent CpGs^[25,26]^. However, elucidating how the density, clustering, and neighboring numbers of methylated CpGs affect 5mC inheritance remains challenging due to the lack of precise manipulation techniques. Our long-strand synthesis approach provides a new opportunity to quantitatively study 5mC dynamics across diverse methylation patterns. As a proof of concept, we studied the influence of 5mC distribution on methylation maintenance at the promoter of a tumor-suppressor gene GSTP1. The GSTP1 promoter, which is sensitive to methylation and typically hypomethylated in normal cells, serves as an ideal genomic locus for investigation of rapid dynamic 5mC behaviors.

Using the aforementioned method, we synthesized five 600 nt ssDNA donors covering the entire GSTP1 promoter region with varying 5mC patterns: (1) non-methylated 600 nt ssDNA (N-5mC, Figure 3b); (2) 1/3–5mC (7/19 CpG sites, with one methylated CpG after two unmethylated CpGs, Figure 3c); (3) 1/2–5mC with even distribution spanning two promoters of GSTP1 (10/19 CpG sites, with one methylated CpG after one unmethylated CpG, Figure 3d); and (4) 2/3–5mC (12/19 CpG sites, two methylated CpGs after one unmethylated CpG, Figure 3e); and (5) F-5mC (19/19 CpG sites, fully methylated, Figure 3f). We use ssDNA as HDR template because ssDNA is reported to enable higher HDR efficiency and less cell toxicity. The purity and 5mC status of these ssDNA donors were characterized by denaturing PAGE and bisulfite sequencing, respectively (Supplementary Figures S12–S13). As illustrated in Figure 3a, prior to ssDNA integration, HEK293T cells with a dual-reporter system containing a split fluorescent protein sfCheery2_1–10_ (fluorescence off) and eGFP (Figure S14) were constructed. After the integration of ssDNA donors into the genome by CRISPR/Cas9-directed HDR, the reactivation of sfCherry2 fluorescence demonstrated the successful homologous recombination of the target ssDNA (Figure S15). The HDR efficiency of the five ssDNA donors ranged from 0.38% to 0.80%, which is relatively lower than previously reported HDR efficiency. This can be attribute to our use of shorter homologous arm (47 nt and 60 nt for the 5’ and 3’ arms, respectively). After two days of post-transfection, genomic DNA from the cells was treated with sodium bisulfite, and the GSTP1 promoter and adjacent regions were amplified using specific primers (Figure S16). The purified amplicons were then subjected to high-throughput sequencing to determine the 5mC ratio at each CpG site, elucidating the maintenance and heritability of methylated sites after ssDNA integration. The 5mC ratio is calculated as the number of unconverted cytosines divided by the total number of cytosines across all mapped reads.

Our results demonstrate that 5mC patterns from the ssDNA donor were precisely transmitted to the native genomic locus, confirming the ability to in-situ manipulation of native genomic 5mC status at single-base resolution by utilizing site-specifically 5mC-modified ssDNA donors. We utilized ssDNA as the HDR template due to its demonstrated higher efficiency in genome editing compared to double-stranded DNA (dsDNA)^[27]^. Upon HDR-mediated integration, the methylated ssDNA creates hemi-methylated CpG sites in the genome (methylated on one strand). During subsequent DNA replication, the native cellular maintenance methyltransferase DNMT1 is anticipated to recognize these hemi-methylated sites and faithfully methylates the complementary cytosines, resulting in fully methylated CpG dinuleotides^[28,29]^. As the CpG island of GSTP1 promoter is hypomethylated (averaged 5mC ratio < 3%, Figure S14c), the observed 10% to 30% is biologically significant, which can be attributed to the successful manipulation of 5mC sites on the native genome and the heritable methylation patterns that are maintained through cell devision cycles^[30]^. Furthermore, we identified a strong positive correlation between the maintenance of 5mC and the density of 5mC sites in parental ssDNA (Figure 3g). This suggests a synergistic effect of 5mC density in counteracting demethylation processes (Figure 3f). Collectively, these findings underscore the potential of our synthesis strategy to manipulate the 5mC landscape and dissect the dynamics of 5mC inheritance as well as its regulatory roles at specific genomic loci.

## Conclusion

The accurate synthesis of long DNA sequences with high fidelity remains a formidable challenge, particular when dealing with high GC contents, repetitive elements, and specific chemical modifications. While enzyme-based approaches have been developed for introducing DNA modifications in long-strand DNA, they are limited by the availability of enzymes and the inability to precisely introduce modifications at specific sites. Therefore, the reliable synthesis of long DNA with site-specific modifications is highly demanded for advancing the study of epigenetics and the development of DNA-based therapeutics.

To address these limitations, we have developed a novel strategy leveraging the mechanism of kinetically-interlocked multiple unit strategy^[24]^. This approach enables the polymerization of multiple s-DNAs with pre-disposed modifications and their complementary c-DNAs into the high-molecular-weight assemblies, allowing the formation of long ssDNA with site-specific modifications. Such long ssDNA with site-specific 5mC modifications combined with HDR would provide an efficient tool for manipulating genomic 5mC across a wide range with single-base resolution. CRISPR/Cas9-directed HDR using methylated DNA donors has been reported for manipulating native 5mC status. However, these methods are restricted by the inherent sequence specificity of recombinant methyltransferases, which cannot synthesize DNA templates with modifications at arbitrary positions, thus limiting single-base resolution manipulation capabilities^[22,31,32]^.

In principle, our strategy possesses several inherent advantages: Firstly, the targeted long ssDNA could be synthesized with high purity. While conserving multiple precise modifications, the length gap between the targeted ssDNA and byproducts is at least 20–40 nt (the length of s-DNA). This disparity ensures effective separation and purification through currently available methods. Secondly, the assembly procedure endows such method with self-proofing property to ensure the high fidelity of targeted ssDNA, which originated from the stability discrimination between assemblies with and without mismatches. By tuning the annealing temperature, even single mismatch could be easily discriminated, which has brought the excellent fidelity of the target ssDNA. Thirdly, according to the mechanism of our strategy, we are able to facilely synthesize long ssDNA with various modifications, which could be used in manipulating genomic 5mC at base-resolution. The site-specifically modified ssDNA offers a fabulous way to fine-tune CpG methylation in any desired genomic locus at single-base resolution and enables us to gain a deeper understanding of 5mC dynamics and shed new light on targeted epigenetic therapy. Fourth, the method could also be used to prepare long stretches of DNAs that contain sequences challenging to amplify through enzymatic approaches. The ability to synthesize and assemble long DNAs also could facilitate synthetic biology and information storage. The same strategy could be used to prepare long ssRNA bearing modifications for numerous potential applications in the future.

While our method successfully synthesizes >1000 nt ssDNA with site-specific modifications, several challenges remain to be addressed. First, the current HPLC purification is expensive and labor-intensive. Second, the recovery efficiency needs further optimization, *e.g*., by anion exchange chromatography. Third, synthesis of ultralong ssDNA (>2000 nt) remains challenging due to decreased recovery, assembly, and ligation efficiencies, combined with difficulties in discriminating target ssDNA from by-products. This limitation could potentially be addressed through a stepwise synthesis approach, progressing from long ssDNA intermediates to ultralong products.

Our strategy has made significant progress in the precise manipulation of 5mC sites, but there is still ample room for further improvement and exploration. While precise control of 5mC has been demonstrated within a few hundred base pairs, it can be anticipated the synthesis of ultralong ssDNA with precise site modifications exceeding 4000 nt could also be realized by extending the length of the s-DNA. Therefore, our strategy would provide a universal tool to regulate critical genomic sites across broader ranges. Furthermore, our method can precisely introduce a wider array of modifications at specific sites of long ssDNA, which is important for synthetic biology and epigenetics. For instance, the unnatural base pair, e.g., dNaM-dTPT3 and dNaM-d5SICS, could be precisely introduced on long ssDNA/dsDNA, which will provide a powerful tool for the creation of a wider variety of semi-synthetic organisms^[33,34]^. In addition, other DNA modifications existing in the genome such as 4mC and 6mA could also be introduced^[35–37]^, expanding the toolkit for epigenetic regulation and facilitating the precise interpretation of their functions.

In conclusion, we have developed a robust method for preparing site-specifically modified long ssDNA with high purity and fidelity. By fine-tuning the density of 5mC using our long ssDNA synthesis approach, we are able to dynamically portray the synergic effect of 5mC in the GSTP1 promoter region. We found a pronounced density-dependent effect in 5mC maintenance. We assume that the cooperative density effect might play a fundamental role in shaping 5mC landscapes, thereby influencing the stability of chromosome and the dynamic plasticity of gene expression. The strategy presented in this paper could be readily applied to prepare long DNAs with or without modification for diverse applications in gene synthesis, information storage, and basic investigations of epigenetic pathways.

## Supplementary Material

Supporting Information

Supporting information for this article is given via a link at the end of the document.

The authors have cited additional references within the Supporting Information.^[33, 34]^
