# Supplementary material for: Long Single-Stranded DNA with Site-Specific Modifications to Enable Single-Base-Resolution Epigenetics
Source: Angew Chem Int Ed Engl. Author manuscript; Available in PMC 2026 Sep 24. (PMC13602406; doi:10.1002/anie.202513078)
Supplement: Supplementary file 1 [file NIHMS2209787-supplement-Supporting_Information.docx]

**Supporting Information**

**Long Single-Stranded DNA with Site-Specific Modifications to Enable Single-Base-Resolution Epigenetics**

Chenyou Zhu^1^, Yin Xu^2^, Ziyang Hao^3*^, Yuan Zhuang^1^, Rui Xu^1^, Yufan Pan^1^, Jiezhong Shi^1^, Yujie Li^1^, Yifan Jiang^1^, Tianran Wang^3^, Yuhe R. Yang^4^, Huajie Liu^5^, Dali Han^2^, Yuanchen Dong^6*^, Chuan He^7,8*^, Dongsheng Liu^1,9*^

AUTHOR ADDRESS

^1^Engineering Research Center of Advanced Rare Earth Materials, (Ministry of Education), Department of Chemistry, Tsinghua University, Beijing 100084, China

^2^China National Center for Bioinformation, Beijing Institute of Genomics, Chinese Academy of Sciences, Beijing, 100101, China

^3^School of Pharmaceutical Sciences, Capital Medical University, Beijing, 100069, PR China

^4^CAS Key Laboratory for Biomedical Effects of Nanomaterials & Nanosafety, CAS Key Laboratory of Nanosystem and Hierarchical Fabrication, CAS Center for Excellence in Nanoscience, National Center for Nanoscience and Technology, Beijing, PR China

^5^School of Chemical Science and Engineering, Shanghai Research Institute for Intelligent Autonomous Systems, Key Laboratory of Advanced Civil Engineering Materials of Ministry of Education, Tongji University, Shanghai 200092, China

^6^CAS Key Laboratory of Colloid, Interface and Chemical Thermodynamics, Institute of Chemistry, Chinese Academy of Sciences, No. 2 Zhongguancun Beiyijie, Beijing, 100190, PR China

^7^Department of Chemistry, Department of Biochemistry and Molecular Biology, Institute for Biophysical Dynamics, The University of Chicago, Chicago, IL 60637, USA

^8^Howard Hughes Medical Institute, The University of Chicago, Chicago, IL 60637, USA

^9^Department of Applied Biology and Chemical Technology, The Hong Kong Polytechnic University, Hong Hum, Kowloon, Hong Kong, China

^*^Corresponding authors: zyhao@ccmu.edu.cn (Z. H); dongyc@iccas.ac.cn (Y. D.); chuanhe@uchicago.edu (C. H.); dongsheng.liu@polyu.edu.hk (D. L.)

**Table of Contents**

Materials and Methods

Table S1 Sequence of used oligos

Figure S1 Assembly results of s-DNA and c-DNA with different melting temperature

Figure S2 One-pot synthesis of 100 nt and 200 nt ssDNA

Figure S3 Characterization of concentration insensitivity of synthesizing 100 nt ssDNA

Figure S4 Tolerance of stochiometric ratio of s-DNA and c-DNA during synthesis of 100 nt ssDNA

Figure S5 One-pot synthesis of 140 to 200 nt ssDNA using 20 nt s-DNA

Figure S6 Characterization of the synthesis efficiency under different reaction time

Figure S7 One-pot synthesis of 1228 nt ssDNA. 1228 nt ssDNA was one-pot synthesized

Figure S8 Heatmap of error rate of synthesized 720 nt ssDNA

Figure S9 Synthesis of 360 nt ssDNA with site-specifically phosphorothioate (pt) modifications

Figure S10 Characterization of ssDNA with site-specific 5mC modifications

Figure S11 Synthesis of ssDNA donor with different 5mC modifications

Figure S12 Bisulfite sequencing of synthesized ssDNA donor

Figure S13 Scheme of construction of cell line and flow cytometry and Bisulfite-PCR characterization

Figure S14 Characterization of the integration of ssDNA donor

Figure S15 Characterization of 5mC ratio of each CpG dyad near GSTP1 promotor

**Materials and Methods**

**Online Methods**

**gRNA synthesis and plasmid construction**

CRISPR gRNAs were designed using Benchling software (<http://benchling.com/>). gRNAs (gRNA1: ACTGGTGGCGAAGACTGCGG ACTGGTGGCGAAGACTGCGG; gRNA2: TCGAACTGGGAAATAGACCA)were synthesized by the Engen^®^ sgRNA synthesis kit, *S. pyrogenes* (New England Biolabs) following the manufacturer’s instruction. The ssDNA templates for sgRNA synthesis were purchase from Beijing Xianghong Biotechnology Co., Ltd and Sango Biotechnology. Plasmid insertion for sfCherry2_1-10_ and eGFP was synthesized by Beijing SupraDNA technology, with dsDNA donor cloned into TOPO/TA vector.

**Cell culture**

HEK293T cells were purchased from National Infrastructure of Cell Line Resource and cultured in Dulbecco’s modified Eagle’s medium (DMEM) plus GlutaMAX-I (Gibco), supplemented with 10% (v/v) fetal bovine serum (Gibco, qualified) and 1×pennicillin streptomycin (Gibco). Both the two kinds of cells were incubated, maintained, and passaged at 37 °C with 5% CO_2_.

**Design and Synthesis of long ssDNA with site-specific modification**

The target ssDNA sequence was systematically divided into multiple s-DNA segments, each ranging from 20-80 nucleotides to ensure compatibility with both HPLC purification of individual segments and high-resolution separation during denaturing PAGE purification of the final long ssDNA product. The length of s-DNAs can be equal firstly and adjusted accordingly. The melting temperature (T_m_) of each overlap region between adjacent s-DNA segments was calculated, and segment lengths were adjusted to maintain T_m_ values above 50°C with differences of less than 5°C between all overlap regions. This design ensures uniform assembly stability and efficient kinetic locking during the hybridization process. Then, the desired s-DNA and c-DNA were purchased from Beijing Xianghong Biotechnology Co., Ltd. All the s-DNA and c-DNA were purified by HPLC and the purity was characterized by denaturing PAGE. s-DNA and c-DNA were further dissolved in nuclease-free water (Milli-Q, Millipore) and their concentration was determined by A_260_ using Nanodrop One (Thermo Fischer Scientific). For small-scale synthesis trials, 10 pmol each of s-DNA and c-DNA were mixed. For large-scale synthesis, 1-10 nmol each of s-DNA and c-DNA were used. The mixture was adjusted to 1×TAE-Mg²⁺ buffer (12.5 mM Mg²⁺) by adding 10×TAE-Mg²⁺ buffer (125 mM Mg²⁺). The solution was heated to 95 °C for 5 min, then slowly cooled to 4 °C at a rate of 1 °C/min for annealing. Following annealing, T4 DNA ligase (Thermo Fisher Scientific) and T4 DNA ligase buffer were added according to the manufacturer's instructions. The mixture was incubated at 37 °C for 1 h to produce the target ssDNA.

**Purification of long ssDNA with site-specific modifications**

After ligation, the mixture was separated by 6% denaturing PAGE (Acrylamide : bis-acrylamide = 19:1 with the addition of 7 M Urea) at 50 °C. After appropriate time of electrophoresis, the uppermost gel band was sliced and soaked in nuclease-free water at 4 °C overnight. Then, the supernatant was filtered, desalted, and concentrated by Amicon Ultra Centrifugal Filter Units (10 kDa, Millipore) to obtain the purified long ssDNA.

**Calculation of error rate of synthesized ssDNA**

Synthesized ssDNA was amplified using specific primers with Phusion Plus PCR Master Mix (Thermo Fisher Scientific, F632S). PCR products were purified using the QIAquick PCR Purification Kit (QIAGEN, 28106) according to the manufacturer's instructions. Purified dsDNA was cloned into TOPO-TA/Blunt vectors using the LabpO TOPO-TA/Blunt Universal cloning kit (Lablead, PT0101) following the manufacturer's protocol. After transforming competent cells and plating on ampicillin-containing agar plates, 40 individual subclones were selected, amplified, and subjected to Sanger sequencing. Each subclone was sequenced twice using M13F and M13R primers.

For calculating the single-base error rate of the 720 nt ssDNA, we applied the following criteria to account for Sanger sequencing limitations: In overlapping regions between the two sequencing runs, sites with errors (mismatches, insertions, or deletions) detected in only one run were considered correct, while errors detected in both runs were counted as true errors. Errors in non-overlapping regions were counted as true errors. Insertions and deletions were assigned to the upstream base of the error site. The total number of errors across all 40 subclones was divided by the total number of sequenced bases (40 × 720 bases) to calculate the single-base error rate.

**Electroporation**

HEK293T cells were seeded to reach 60%-80% confluency one day before electroporation. 5 µg Engen^@^ Spy Cas9 NLS (New England Biolabs) and 1.25 µg of synthesized sgRNA were mixed with 50 µL buffer R (Invitrogen) and incubated at room temperature for 15 min to form ribonucleoprotein complex (RNP). At the same time, cells were harvested and counted. 5×10^5^ cells were washed by DPBS (Gibco) twice and resuspended in 50 µL buffer R. The Cas9-sgRNA ribonucleoprotein solution and cell suspension were then mixed, and the appropriate amount of donor DNA was then added. Cells were electroporated using Neon Transfection System (Invitrogen) according to the manufacturer’s instruction. After that, cells were transferred into a prewarmed complete medium without antibodies and cultured for several days.

**Cell sorting and flow cytometry**

The eGFP-positive 293T cells were sorted into 15 mL tubes with a complete medium. Cells were further centrifuged and cultured in a complete medium with 1×pennicillin streptomycin. For flow cytometry analysis, cells were dissociated by TrypLE (Gibco) resuspended in PBS (Gibco), and analyzed with Aria SORP (Becton Dickinson) under FITC and PE channels.

**Genomic DNA isolation and bisulfite conversion**

Desired cells were cultured and harvested. Genomic DNA was extracted using Purelink Genomic DNA Kits (Invitrogen) following the manufacturer’s instructions. Denaturation and bisulfite conversion of genomic DNA was conducted using EZ DNA Methylation-Gold Kit (Zymo Research) following the manufacturer’s instruction. The bisulfite conversion product was eluted using 10 µL nuclease-free water which was provided in the kit, and 1 µL of it was amplified using Platinum II Taq Hot-Start DNA Polymerase (Invitrogen) with specific primers. The PCR products were characterized by 1.5% agarose gel electrophoresis and the target band was excised and purified using QIAquick Gel Extraction Kit (QIAGEN) following the manufacturer’s instruction. The purified products were used for Sanger sequencing or Next-generation sequencing.

**TA cloning and Sanger sequencing**

PCR products were subcloned into TOPO/TA vector using LabPO TOPO-TA/Blunt Cloning Kit (Lablead) and DH5𝛼 competent E. Coli. Then single positive colonies were picked and sequenced using Sanger sequencing with ABI 3730xl sequencer.

**High-throughput DNA sequencing of targeted methylation sites**

Genomic sites of interest were bisulfite-conversed, amplified from genomic DNA samples, and sequenced on an Illumina Novaseq platform. In brief, the genomic DNA of cells of interest was extracted and bisulfite-conversed. Targeted sites of interest were amplified using specific primers, and PCR products were purified by electrophoresis in a 1.5% agarose gel. Purified PCR products were fragmented using Bioruptor Pico Sonication System (diagenode), and the adaptor sequences and barcodes were added using Hieff NGS^TM^ Ultima Pro DNA Library Prep Kit for Illumina V2 (Yeasen Biotechnology) with Hieff NGS^TM^ 384 CDI Primer for Illumina, Set 1 (Yeasen Biotechnology) following the manufacturer’s instructions. The purified PCR products containing the adaptors and barcodes were run on Illumina Novaseq (150×150-paired end read).

**Data processing and calling of methylation ratio of individual CpG sites**

Raw bisulfite sequencing data were pre-processed by fastp version 0.12.4 (https://github.com/OpenGene/fastp) with command "fastp -i R1.fastq -I R2.fastq -o R1.out.fastq -O R2.out.fastq -h A.html -j B.html", which included adapter trimming, removing the reads in which the N base has reached a certain percentage (default length of 5 bp), removing reads which contain low quality bases (threshold value≤20) above 40%, and sliding window trimming^[33]^. Quality Control on adapter-free reads were performed using FastQC v0.11.9 (https://www.bioinformatics.babraham.ac.uk/projects/fastqc/). An index for the reference oligonucleotides sequence was constructed using the function “bismark_genome_preparation” from the Bismark version 0.24.0 (https://github.com/FelixKrueger/Bismark)^[34]^. Alignment was then performed using "bismark --bowtie2 --un --samtools_path -o output_path reference_index -1 R1.fastq -2 R2.fastq". The output bam files were deduplicated using "deduplicate_bismark -p R1_bismark_bt2_pe.bam --bam --output_dir". Finally, methylation information was extracted using the function“bismark_methylation_extractor” with command "bismark_methylation_extractor -s --gzip --comprehensive -bedGraph --counts --report --cytosine_report --genome_folder reference_index R1_bismark_bt2_pe.deduplicated.bam -o output_path". Methylation values at each site were calculated as total (forward and reverse) non-converted C-reads over total (forward and reverse) reads. CpGs were counted once per location combining both strands together.

**Table S1.** Sequence of used oligos

X denotes the 5mC modification site. 5’-P denotes 5’-phosphate modification. Mismatched bases are marked in red.

| Name | Sequence (5’-3’) | Length (nt) | Modification |
| --- | --- | --- | --- |
| 100 nt-s-1 | GTCTGCTCCTGTCGCCTTCG | 20 | - |
| 100 nt-s-2 | CCTCCTAATCACTAGCCACT | 20 | 5’-P |
| 100 nt-s-3 | ATGGAACACGTGGCCTTCGG | 20 | 5’-P |
| 100 nt-s-4 | CAGCGAGGACATCGAGAACA | 20 | 5’-P |
| 100 nt-s-5 | CCCTGGCCAAGATGGACGAC | 20 | 5’-P |
| 100 nt-c-4 | TTGGCCAGGGTGTTCTCGAT | 20 | - |
| 100 nt-c-3 | GTCCTCGCTGCCGAAGGCCA | 20 | - |
| 100 nt-c-2 | CGTGTTCCATAGTGGCTAGT | 20 | - |
| 100 nt-c-1 | GATTAGGAGGCGAAGGCGAC | 20 | - |
| 200 nt-s-1 | GTCTGCTCCTGTCGCCTTCG | 20 | - |
| 200 nt-s-1-cy3 | GTCTGCTCCTGTCGCCTTCG | 20 | 5’-cy3 |
| 200 nt-s-2 | CCTCCTAATCACTAGCCACT | 20 | 5’-P |
| 200 nt-s-3 | ATGGAACACGTGGCCTTCGG | 20 | 5’-P |
| 200 nt-s-4 | CAGCGAGGACATCGAGAACA | 20 | 5’-P |
| 200 nt-s-5 | CCCTGGCCAAGATGGACGAC | 20 | 5’-P |
| 200 nt-s-6 | GGCCAGCTGGACGGACTGGC | 20 | 5’-P |
| 200 nt-s-7 | TTTTGGCGCCATTCAGCTGG | 20 | 5’-P |
| 200 nt-s-8 | ATGGCGATGGAAATATCCTG | 20 | 5’-P |
| 200 nt-s-9 | CAATACAACGCCGCCGAGGG | 20 | 5’-P |
| 200 nt-s-10 | AGATATCACCGGCAGAGATC | 20 | 5’-P |
| 200 nt-c-9 | GGTGATATCTCCCTCGGCGG | 20 | - |
| 200 nt-c-8 | CGTTGTATTGCAGGATATTT | 20 | - |
| 200 nt-c-7 | CCATCGCCATCCAGCTGAAT | 20 | - |
| 200 nt-c-6 | GGCGCCAAAAGCCAGTCCGT | 20 | - |
| 200 nt-c-5 | CCAGCTGGCCGTCGTCCATC | 20 | - |
| 200 nt-c-4 | TTGGCCAGGGTGTTCTCGAT | 20 | - |
| 200 nt-c-3 | GTCCTCGCTGCCGAAGGCCA | 20 | - |
| 200 nt-c-2 | CGTGTTCCATAGTGGCTAGT | 20 | - |
| 200 nt-c-1 | GATTAGGAGGCGAAGGCGAC | 20 | - |
| S-3 | TAACTACTTGTCACTCTCTT | 20 | 3'-FAM |
| M1A | TAACTACTTGTCACTCTCTA | 20 | 3'-Cy5.5 |
| M1B | TAACTACTTGTCACTGTCTT | 20 | 3'-Cy5.5 |
| M1C | TAACTACTTGTGACTCTCTT | 20 | 3'-Cy5.5 |
| M2A | TAACAACTTGTCACTGTCTT | 20 | 3'-Cy5.5 |
| M2B | TAACTACTTGTCAGTCTGTT | 20 | 3'-Cy5.5 |
| M3A | TATCTAGTTGTCACTGTCTT | 20 | 3'-Cy5.5 |
| 720 nt-1-s-1 | TTGCCTTTTATGGTAATAACGCGGCCGGCCCGGCTTCCTT | 40 | - |
| 720 nt-1-s-1-cy3 | TTGCCTTTTATGGTAATAACGCGGCCGGCCCGGCTTCCTT | 40 | 5’-cy3 |
| 720 nt-1-s-2 | TGTCCCCAATCTGGGCGCGCGCCGGCGCCCCCTGGCGGCC | 40 | 5’-P |
| 720 nt-1-s-3 | TAAGGACTCGGCGCGCCGGAAGTGGCCAGGGCGGGGGCGA | 40 | 5’-P |
| 720 nt-1-s-4 | CCTCGGCTCACAGCGCGCCCGGCTATTCTCGCAACTGACA | 40 | 5’-P |
| 720 nt-1-s-5 | ATGGAACACGTGGCCTTCGGCAGCGAGGACATCGAGAACA | 40 | 5’-P |
| 720 nt-1-s-6 | CCCTGGCCAAGATGGACGACGGCCAGCTGGACGGACTGGC | 40 | 5’-P |
| 720 nt-1-s-7 | TTTTGGCGCCATTCAGCTGGATGGCGATGGAAATATCCTG | 40 | 5’-P |
| 720 nt-1-s-8 | CAATACAACGCCGCCGAGGGAGATATCACCGGCAGAGATC | 40 | 5’-P |
| 720 nt-1-s-9 | CTAAGCAGGTGATCGGCAAGAACTTCTTCAAGGACGTGGC | 40 | 5’-P |
| 720 nt-1-s-10 | CCCTGGCACCGACTCCCCAGAATTCTACGGCAAGTTCAAG | 40 | 5’-P |
| 720 nt-1-s-11 | GAAGGCGTGGCTTCTGGCAACCTGAACACCATGTTCGAGT | 40 | 5’-P |
| 720 nt-1-s-12 | GGATGATCCCCACAAGCCGGGGCCCTACAAAGGTGAAGGT | 40 | 5’-P |
| 720 nt-1-s-13 | GCACATGAAAAAGGCCCTGAGCGGCGACAGCTACTGGGTC | 40 | 5’-P |
| 720 nt-1-s-14 | TTTGTGAAAAGAGTGGCCGGCTCCGGTACCGATGATGATA | 40 | 5’-P |
| 720 nt-1-s-15 | TCGCAGCGCTCGTCGTCGACAACGGCTCCGGCATGTGCAA | 40 | 5’-P |
| 720 nt-1-s-16 | GGCCGGCTTCGCGGGCGACGATGCCCCCCGGGCCGTCTTC | 40 | 5’-P |
| 720 nt-1-s-17 | CCCTCCATCGTGGGGCGCCCCAGGCACCAGGTAGGGGAGC | 40 | 5’-P |
| 720 nt-1-s-18 | TGGCTGGGTGGGGCAGCCCCGGGAGCGGGCGGGAGGCAAG | 40 | 5’-P |
| 720 nt-1-c-17 | GGGGCTGCCCCACCCAGCCAGCTCCCCTACCTGGTGCCTG | 40 | - |
| 720 nt-1-c-16 | GGGCGCCCCACGATGGAGGGGAAGACGGCCCGGGGGGCAT | 40 | - |
| 720 nt-1-c-15 | CGTCGCCCGCGAAGCCGGCCTTGCACATGCCGGAGCCGTT | 40 | - |
| 720 nt-1-c-14 | GTCGACGACGAGCGCTGCGATATCATCATCGGTACCGGAG | 40 | - |
| 720 nt-1-c-13 | CCGGCCACTCTTTTCACAAAGACCCAGTAGCTGTCGCCGC | 40 | - |
| 720 nt-1-c-12 | TCAGGGCCTTTTTCATGTGCACCTTCACCTTTGTAGGGCC | 40 | - |
| 720 nt-1-c-11 | CCGGCTTGTGGGGATCATCCACTCGAACATGGTGTTCAGG | 40 | - |
| 720 nt-1-c-10 | TTGCCAGAAGCCACGCCTTCCTTGAACTTGCCGTAGAATT | 40 | - |
| 720 nt-1-c-9 | CTGGGGAGTCGGTGCCAGGGGCCACGTCCTTGAAGAAGTT | 40 | - |
| 720 nt-1-c-8 | CTTGCCGATCACCTGCTTAGGATCTCTGCCGGTGATATCT | 40 | - |
| 720 nt-1-c-7 | CCCTCGGCGGCGTTGTATTGCAGGATATTTCCATCGCCAT | 40 | - |
| 720 nt-1-c-6 | CCAGCTGAATGGCGCCAAAAGCCAGTCCGTCCAGCTGGCC | 40 | - |
| 720 nt-1-c-5 | GTCGTCCATCTTGGCCAGGGTGTTCTCGATGTCCTCGCTG | 40 | - |
| 720 nt-1-c-4 | CCGAAGGCCACGTGTTCCATTGTCAGTTGCGAGAATAGCC | 40 | - |
| 720 nt-1-c-3 | GGGCGCGCTGTGAGCCGAGGTCGCCCCCGCCCTGGCCACT | 40 | - |
| 720 nt-1-c-2 | TCCGGCGCGCCGAGTCCTTAGGCCGCCAGGGGGCGCCGGC | 40 | - |
| 720 nt-1-c-1 | GCGCGCCCAGATTGGGGACAAAGGAAGCCGGGCCGGCCGC | 40 | - |
| 720 nt-2-s-1 | CTAGGCTGCAGTGAGCCTAGATGGCATCACTGCACTCAAG | 40 | - |
| 720 nt-2-s-2 | CCTGGGCGACAGACCTTATCTCTAAAAAAATAAAGATTGC | 40 | 5’-P |
| 720 nt-2-s-3 | ATGAGTATTTTGTTCCACTTGACAGTCATCAATAGATTGG | 40 | 5’-P |
| 720 nt-2-s-4 | TTTAAATTGTGATATCTTTTTTACTTCCCGCAGATGGAAC | 40 | 5’-P |
| 720 nt-2-s-5 | ACGTGGCCTTCGGCAGCGAGGACATCGAGAACACCCTGGC | 40 | 5’-P |
| 720 nt-2-s-6 | CAAGATGGACGACGGCCAGCTGGACGGACTGGCTTTTGGC | 40 | 5’-P |
| 720 nt-2-s-7 | GCCATTCAGCTGGATGGCGATGGAAATATCCTGCAATACA | 40 | 5’-P |
| 720 nt-2-s-8 | ACGCCGCCGAGGGAGATATCACCGGCAGAGATCCTAAGCA | 40 | 5’-P |
| 720 nt-2-s-9 | GGTGATCGGCAAGAACTTCTTCAAGGACGTGGCCCCTGGC | 40 | 5’-P |
| 720 nt-2-s-10 | ACCGACTCCCCAGAATTCTACGGCAAGTTCAAGGAAGGCG | 40 | 5’-P |
| 720 nt-2-s-11 | TGGCTTCTGGCAACCTGAACACCATGTTCGAGTGGATGAT | 40 | 5’-P |
| 720 nt-2-s-12 | CCCCACAAGCCGGGGCCCTACAAAGGTGAAGGTGCACATG | 40 | 5’-P |
| 720 nt-2-s-13 | AAAAAGGCCCTGAGCGGCGACAGCTACTGGGTCTTTGTGA | 40 | 5’-P |
| 720 nt-2-s-14 | AAAGAGTGGGAGGCTCTGGAGGCTCTCGTGAGTGCATCTC | 40 | 5’-P |
| 720 nt-2-s-15 | CATCCACGTTGGCCAGGCTGGTGTCCAGATTGGCAATGCC | 40 | 5’-P |
| 720 nt-2-s-16 | TGCTGGGAGCTCTACTGCCTGGAACACGGCATCCAGCCCG | 40 | 5’-P |
| 720 nt-2-s-17 | ATGGCCAGATGCCAAGTGACAAGACCATTGGGGGAGGAGA | 40 | 5’-P |
| 720 nt-2-s-18 | TGACTCCTTCAACACCTTCTTCAGTGAGACGGGCGCTGGC | 40 | 5’-P |
| 720 nt-2-c-17 | GATAAGGTCTGTCGCCCAGGCTTGAGTGCAGTGATGCCAT | 40 | - |
| 720 nt-2-c-16 | AAGTGGAACAAAATACTCATGCAATCTTTATTTTTTTAGA | 40 | - |
| 720 nt-2-c-15 | AAAAGATATCACAATTTAAACCAATCTATTGATGACTGTC | 40 | - |
| 720 nt-2-c-14 | CTCGCTGCCGAAGGCCACGTGTTCCATCTGCGGGAAGTAA | 40 | - |
| 720 nt-2-c-13 | GCTGGCCGTCGTCCATCTTGGCCAGGGTGTTCTCGATGTC | 40 | - |
| 720 nt-2-c-12 | TCGCCATCCAGCTGAATGGCGCCAAAAGCCAGTCCGTCCA | 40 | - |
| 720 nt-2-c-11 | GATATCTCCCTCGGCGGCGTTGTATTGCAGGATATTTCCA | 40 | - |
| 720 nt-2-c-10 | AGAAGTTCTTGCCGATCACCTGCTTAGGATCTCTGCCGGT | 40 | - |
| 720 nt-2-c-9 | TAGAATTCTGGGGAGTCGGTGCCAGGGGCCACGTCCTTGA | 40 | - |
| 720 nt-2-c-8 | GTTCAGGTTGCCAGAAGCCACGCCTTCCTTGAACTTGCCG | 40 | - |
| 720 nt-2-c-7 | TAGGGCCCCGGCTTGTGGGGATCATCCACTCGAACATGGT | 40 | - |
| 720 nt-2-c-6 | TCGCCGCTCAGGGCCTTTTTCATGTGCACCTTCACCTTTG | 40 | - |
| 720 nt-2-c-5 | TCCAGAGCCTCCCACTCTTTTCACAAAGACCCAGTAGCTG | 40 | - |
| 720 nt-2-c-4 | CAGCCTGGCCAACGTGGATGGAGATGCACTCACGAGAGCC | 40 | - |
| 720 nt-2-c-3 | AGGCAGTAGAGCTCCCAGCAGGCATTGCCAATCTGGACAC | 40 | - |
| 720 nt-2-c-2 | GTCACTTGGCATCTGGCCATCGGGCTGGATGCCGTGTTCC | 40 | - |
| 720 nt-2-c-1 | AGAAGGTGTTGAAGGAGTCATCTCCTCCCCCAATGGTCTT | 40 | - |
| 720 nt-3-s-1 | GATTCAATTCTTTGAATACAAAGAGCATTTTATTTTGAAG | 40 | - |
| 720 nt-3-s-2 | TTAGAATACTAATTAAATGCTTATGACACTTTAAAAAATT | 40 | 5’-P |
| 720 nt-3-s-3 | ATTTTTTTTTTCTTTCAGAGAATTGTAAGTGCTCAGAGCT | 40 | 5’-P |
| 720 nt-3-s-4 | TGGCTGAAGATGATGTGGAAGGCGGTAGCGGGGATCCACC | 40 | 5’-P |
| 720 nt-3-s-5 | GGTCGCCACCATGGAACACGTGGCCTTCGGCAGCGAGGAC | 40 | 5’-P |
| 720 nt-3-s-6 | ATCGAGAACACCCTGGCCAAGATGGACGACGGCCAGCTGG | 40 | 5’-P |
| 720 nt-3-s-7 | ACGGACTGGCTTTTGGCGCCATTCAGCTGGATGGCGATGG | 40 | 5’-P |
| 720 nt-3-s-8 | AAATATCCTGCAATACAACGCCGCCGAGGGAGATATCACC | 40 | 5’-P |
| 720 nt-3-s-9 | GGCAGAGATCCTAAGCAGGTGATCGGCAAGAACTTCTTCA | 40 | 5’-P |
| 720 nt-3-s-10 | AGGACGTGGCCCCTGGCACCGACTCCCCAGAATTCTACGG | 40 | 5’-P |
| 720 nt-3-s-11 | CAAGTTCAAGGAAGGCGTGGCTTCTGGCAACCTGAACACC | 40 | 5’-P |
| 720 nt-3-s-12 | ATGTTCGAGTGGATGATCCCCACAAGCCGGGGCCCTACAA | 40 | 5’-P |
| 720 nt-3-s-13 | AGGTGAAGGTGCACATGAAAAAGGCCCTGAGCGGCGACAG | 40 | 5’-P |
| 720 nt-3-s-14 | CTACTGGGTCTTTGTGAAAAGAGTGTAATGAGAAACAAAT | 40 | 5’-P |
| 720 nt-3-s-15 | GTCAACATAATAAAATCTCAGTTAAAAATATTTTAAAAAT | 40 | 5’-P |
| 720 nt-3-s-16 | TCTTGGTAGTTGAGCAGCTCTGGGGTAATAAGGGCAAATA | 40 | 5’-P |
| 720 nt-3-s-17 | TGCTTGTTATGAACTACACTGAAATCTACCAAAGTTAATG | 40 | 5’-P |
| 720 nt-3-s-18 | TTTACTTTGTGTAGATCCATTTGTCTATTTTATTTATTTT | 40 | 5’-P |
| 720 nt-3-c-17 | ATGGATCTACACAAAGTAAACATTAACTTTGGTAGATTTC | 40 | - |
| 720 nt-3-c-16 | AGTGTAGTTCATAACAAGCATATTTGCCCTTATTACCCCA | 40 | - |
| 720 nt-3-c-15 | GAGCTGCTCAACTACCAAGAATTTTTAAAATATTTTTAAC | 40 | - |
| 720 nt-3-c-14 | TGAGATTTTATTATGTTGACATTTGTTTCTCATTACACTC | 40 | - |
| 720 nt-3-c-13 | TTTTCACAAAGACCCAGTAGCTGTCGCCGCTCAGGGCCTT | 40 | - |
| 720 nt-3-c-12 | TTTCATGTGCACCTTCACCTTTGTAGGGCCCCGGCTTGTG | 40 | - |
| 720 nt-3-c-11 | GGGATCATCCACTCGAACATGGTGTTCAGGTTGCCAGAAG | 40 | - |
| 720 nt-3-c-10 | CCACGCCTTCCTTGAACTTGCCGTAGAATTCTGGGGAGTC | 40 | - |
| 720 nt-3-c-9 | GGTGCCAGGGGCCACGTCCTTGAAGAAGTTCTTGCCGATC | 40 | - |
| 720 nt-3-c-8 | ACCTGCTTAGGATCTCTGCCGGTGATATCTCCCTCGGCGG | 40 | - |
| 720 nt-3-c-7 | CGTTGTATTGCAGGATATTTCCATCGCCATCCAGCTGAAT | 40 | - |
| 720 nt-3-c-6 | GGCGCCAAAAGCCAGTCCGTCCAGCTGGCCGTCGTCCATC | 40 | - |
| 720 nt-3-c-5 | TTGGCCAGGGTGTTCTCGATGTCCTCGCTGCCGAAGGCCA | 40 | - |
| 720 nt-3-c-4 | CGTGTTCCATGGTGGCGACCGGTGGATCCCCGCTACCGCC | 40 | - |
| 720 nt-3-c-3 | TTCCACATCATCTTCAGCCAAGGACTGTGCACTTACAATT | 40 | - |
| 720 nt-3-c-2 | CTCTGAAAGAAAAAAAAAATAATTTTTTAAAGTGTCATAA | 40 | - |
| 720 nt-3-c-1 | GCATTTAATTAGTATTCTAACTTCAAAATAAAATGCTCTT | 40 | - |
| 614 nt-1-s-1 | AGTGTAGGTAGGGGGTATAGGTTTTTGAGTTGTATTTATT | 40 | - |
| 614 nt-1-s-2 | AAAGTGTGTAGAAGTTGATGGAATAGTGGGGGTAAAGTTA | 40 | 5’-P |
| 614 nt-1-s-3 | TTGTGTTAGTGATGTTATGAGATGGTTATCAGAGTTTCAT | 40 | 5’-P |
| 614 nt-1-s-4 | GTCGCCAACGGAATACTTACCACAGGAGGGAGCACATACA | 40 | 5’-P |
| 614 nt-1-s-5 | CTGAGGCGCGCCGTCGGCCTCTGTTCAAAGGACATTGGTC | 40 | 5’-P |
| 614 nt-1-s-6 | ATCCTATCCATGAAACGTATGGGGTCGAAATAAAAAGACA | 40 | 5’-P |
| 614 nt-1-s-7 | GTATATCTAATGATGTTAGGTATCGTACAGATGAGTGATA | 40 | 5’-P |
| 614 nt-1-s-8 | CCGCGTTACGACCAGAATTTGCTGGCTCTCAGGTGCTCAA | 40 | 5’-P |
| 614 nt-1-s-9 | CTTTACATGGAGAAAACACCTTCTCCCGTTTATGACAAGG | 40 | 5’-P |
| 614 nt-1-s-10 | TTCGGGGGATCTTGACTATACGTGAGCCCCCGAACAAACC | 40 | 5’-P |
| 614 nt-1-s-11 | ATCGACTCTAGCATCACCAAATTGCCACTACAACATCTGC | 40 | 5’-P |
| 614 nt-1-s-12 | TTATAGAACTGGGAAGGGTAGGGCTAGGATATGCATAGGC | 40 | 5’-P |
| 614 nt-1-s-13 | AATATTAGACCTGTAACGCTCCTCGCAAGCCATAAGGCTG | 40 | 5’-P |
| 614 nt-1-s-14 | CGAATGGCCCAAGAGGTCCAATGCCCGCTTGCACGATTTC | 40 | 5’-P |
| 614 nt-1-s-15 | CGAGGAACGAGCGACGTCAGCTGTCCGGCTTCGAGATTGAACCCTGACGGGTTG | 54 | 5’-P |
| 614 nt-1-c-14 | CATCAACTTCTACACACTTTAATAAATACAACTCAAAAAC | 40 | - |
| 614 nt-1-c-13 | TCATAACATCACTAACACAATAACTTTACCCCCACTATTC | 40 | - |
| 614 nt-1-c-12 | GTAAGTATTCCGTTGGCGACATGAAACTCTGATAACCATC | 40 | - |
| 614 nt-1-c-11 | AGGCCGACGGCGCGCCTCAGTGTATGTGCTCCCTCCTGTG | 40 | - |
| 614 nt-1-c-10 | ATACGTTTCATGGATAGGATGACCAATGTCCTTTGAACAG | 40 | - |
| 614 nt-1-c-9 | CCTAACATCATTAGATATACTGTCTTTTTATTTCGACCCC | 40 | - |
| 614 nt-1-c-8 | AAATTCTGGTCGTAACGCGGTATCACTCATCTGTACGATA | 40 | - |
| 614 nt-1-c-7 | GGTGTTTTCTCCATGTAAAGTTGAGCACCTGAGAGCCAGC | 40 | - |
| 614 nt-1-c-6 | TATAGTCAAGATCCCCCGAACCTTGTCATAAACGGGAGAA | 40 | - |
| 614 nt-1-c-5 | TTGGTGATGCTAGAGTCGATGGTTTGTTCGGGGGCTCACG | 40 | - |
| 614 nt-1-c-4 | TACCCTTCCCAGTTCTATAAGCAGATGTTGTAGTGGCAAT | 40 | - |
| 614 nt-1-c-3 | AGCGTTACAGGTCTAATATTGCCTATGCATATCCTAGCCC | 40 | - |
| 614 nt-1-c-2 | TGGACCTCTTGGGCCATTCGCAGCCTTATGGCTTGCGAGG | 40 | - |
| 614 nt-1-c-1 | CTGACGTCGCTCGTTCCTCGGAAATCGTGCAAGCGGGCAT | 40 | - |
| 614 nt-2-s-1 | TGGGCGTAGATCATTCGCTAAACTAACTCGGACGCAGGGG | 40 | - |
| 614 nt-2-s-2 | CACCCAGGCCAGCGTGCAGACGAAGTAGTCGTCTACCCCA | 40 | 5’-P |
| 614 nt-2-s-3 | TTTTCGTGTTTGAGAGACTGCAACCGGTTTTTAAGCAGTC | 40 | 5’-P |
| 614 nt-2-s-4 | CCCTTGGGTGGAAAGCGGAGTAATAGTTGGCAGCAAAATG | 40 | 5’-P |
| 614 nt-2-s-5 | TAGCCGATCCGCATGCGCACTTCACTCCGTGGTGACCGTA | 40 | 5’-P |
| 614 nt-2-s-6 | AAGTTCCACGGCGAGTCTGATCGCGATGCTATGTGAATCC | 40 | 5’-P |
| 614 nt-2-s-7 | CACCGACACGTTGCGGCATTAACAGCCTAAGTCAATTACT | 40 | 5’-P |
| 614 nt-2-s-8 | CACACAATCTCTTTGTTGATTATTCTATTGTCACGCGGTA | 40 | 5’-P |
| 614 nt-2-s-9 | CCTACGCCCTAGAGCTTTTGGACTTGTATTTAGTACGGTG | 40 | 5’-P |
| 614 nt-2-s-10 | TGTACTAGTGGCGGGCCGCCTGCCGGGACCCGGATTCAGT | 40 | 5’-P |
| 614 nt-2-s-11 | GCGTCCTGGTAAGAAGCTACTGTGCCTTTCTTCCTTAGCG | 40 | 5’-P |
| 614 nt-2-s-12 | CTGAAGATAATCGGTCTTAATTCCCTCATATAACCTCCAG | 40 | 5’-P |
| 614 nt-2-s-13 | TTAAATCAAGTGTCTCGATAGCTCGTTCTGGATGAGGTAG | 40 | 5’-P |
| 614 nt-2-s-14 | TGTTGGTTTATTGAATAGGTGTGAAGAAAATATTTGTTAA | 40 | 5’-P |
| 614 nt-2-s-15 | TTAGGTGATATAAAGTAGAGTTAAATAAAGTGTATGGATAGGTGGTTGTGTATG | 54 | 5’-P |
| 614 nt-2-c-14 | TCTGCACGCTGGCCTGGGTGCCCCTGCGTCCGAGTTAGTT | 40 | - |
| 614 nt-2-c-13 | CAGTCTCTCAAACACGAAAATGGGGTAGACGACTACTTCG | 40 | - |
| 614 nt-2-c-12 | CTCCGCTTTCCACCCAAGGGGACTGCTTAAAAACCGGTTG | 40 | - |
| 614 nt-2-c-11 | GTGCGCATGCGGATCGGCTACATTTTGCTGCCAACTATTA | 40 | - |
| 614 nt-2-c-10 | TCAGACTCGCCGTGGAACTTTACGGTCACCACGGAGTGAA | 40 | - |
| 614 nt-2-c-9 | AATGCCGCAACGTGTCGGTGGGATTCACATAGCATCGCGA | 40 | - |
| 614 nt-2-c-8 | ATCAACAAAGAGATTGTGTGAGTAATTGACTTAGGCTGTT | 40 | - |
| 614 nt-2-c-7 | CAAAAGCTCTAGGGCGTAGGTACCGCGTGACAATAGAATA | 40 | - |
| 614 nt-2-c-6 | GGCGGCCCGCCACTAGTACACACCGTACTAAATACAAGTC | 40 | - |
| 614 nt-2-c-5 | GTAGCTTCTTACCAGGACGCACTGAATCCGGGTCCCGGCA | 40 | - |
| 614 nt-2-c-4 | TTAAGACCGATTATCTTCAGCGCTAAGGAAGAAAGGCACA | 40 | - |
| 614 nt-2-c-3 | TATCGAGACACTTGATTTAACTGGAGGTTATATGAGGGAA | 40 | - |
| 614 nt-2-c-2 | ACCTATTCAATAAACCAACACTACCTCATCCAGAACGAGC | 40 | - |
| 614 nt-2-c-1 | CTCTACTTTATATCACCTAATTAACAAATATTTTCTTCAC | 40 | - |
| 720 nt-1-s-1-pt | T*T*G*CCTTTTATGGTAATAACGCGGCCGGCCCGGCTTCCTT | 40 | phosphorothioate (pt) |
| 720 nt-1-s-3-pt | TAAGGACTCGG*CGCGCCGGAAGTGGCCAGGGCGGGGGCGA | 40 | 5’-P, pt |
| 720 nt-1-s-9-pt | CTAAGCAGGTGATCGGCAAGAACTTCTTCAAGGACGT*G*G*C | 40 | 5’-P, pt |
| 720 nt-2-s-6-FAM | CAAGATGGACGACGGCCAGCTGGACGGACTGGCTTTTGGC | 40 | 5’-P, dT-FAM |
| 720 nt-2-s-7-cy5 | GCCATTCAGCTGGATGGCGATGGAAATATCCTGCAATACA | 40 | 5’-P, dT-cy5 (marked with red colour) |
| 500 nt-s-1 | AGGGAGTTGATTTCAGACTATGCACCAGATTGTTTTGTGTTAATAGAAAT | 50 | - |
| 500 nt-s-2 | GAATGGCAAGTTAGTCAAAACTACGATTGAAGAACTCATTCCACTCAAAT | 50 | 5’-P |
| 500 nt-s-3 | ATACCCGAAATTAGTTAATTAAGAATTCGTCCCACCGCGATGTCCCGGCG | 50 | 5’-P |
| 500 nt-s-4 | CGCCAGTTCGCTGCGCACACTTCGCTGCGGTCCTCTTCCTGCTGTCTGTT | 50 | 5’-P |
| 500 nt-s-5 | TACTCCCTAGGCCCCGCTGGGGACCTGGGAAAGAGGGAAAGGCTTCCCCG | 50 | 5’-P |
| 500 nt-s-6 | GCCAGCTGCGCGGCGACTCCGGGGACTCCAGGGCGCCCCTCTGCGGCCGA | 50 | 5’-P |
| 500 nt-s-7 | CGCCCGGGGTGCAGCGGCCGCCGGGGCTGGGGCCGGCGGGAGTCCGCGGG | 50 | 5’-P |
| 500 nt-s-8 | ACCCTCCAGAAGAGCGGCCGGCGCCGTGACTCAGCACTGGGGCGGAGCGG | 50 | 5’-P |
| 500 nt-s-9 | GGCGGGACCACCCTTATAAGGCTCGGAGGCCGCGAGGCCTTCGCTGGAGT | 50 | 5’-P |
| 500 nt-s-10 | TTCGCAGCCGCAGTCTTCGCCACCAGTGAGTACGCGCGGCCCGCGTCCCC | 50 | 5’-P |
| 500 nt-c-9 | TGGTGGCGAAGACTGCGGCTGCGAAACTCCAGCGAAGGCCTCGCGGCCTC | 50 | - |
| 500 nt-c-8 | CGAGCCTTATAAGGGTGGTCCCGCCCCGCTCCGCCCCAGTGCTGAGTCAC | 50 | - |
| 500 nt-c-7 | GGCGCCGGCCGCTCTTCTGGAGGGTCCCGCGGACTCCCGCCGGCCCCAGC | 50 | - |
| 500 nt-c-6 | CCCGGCGGCCGCTGCACCCCGGGCGTCGGCCGCAGAGGGGCGCCCTGGAG | 50 | - |
| 500 nt-c-5 | TCCCCGGAGTCGCCGCGCAGCTGGCCGGGGAAGCCTTTCCCTCTTTCCCA | 50 | - |
| 500 nt-c-4 | GGTCCCCAGCGGGGCCTAGGGAGTAAACAGACAGCAGGAAGAGGACCGCA | 50 | - |
| 500 nt-c-3 | GCGAAGTGTGCGCAGCGAACTGGCGCGCCGGGACATCGCGGTGGGACGAA | 50 | - |
| 500 nt-c-2 | TTCTTAATTAACTAATTTCGGGTATATTTGAGTGGAATGAGTTCTTCAAT | 50 | - |
| 500 nt-c-1 | CGTAGTTTTGACTAACTTGCCATTCATTTCTATTAACACAAAACAATCTG | 50 | - |
| 500 nt-s-6-5mC | GCCAGCTGXGXGGXGACTCXGGGGACTCCAGGGXGCCCCTCTGXGGCXGA | 50 | 5’-P, 5mC |
| 500 nt-s-7-5mC | XGCCXGGGGTGCAGXGGCXGCXGGGGCTGGGGCXGGXGGGAGTCXGXGGG | 50 | 5’-P, 5mC |
| 500 nt-s-8-5mC | ACCCTCCAGAAGAGXGGCXGGXGCXGTGACTCAGCACTGGGGXGGAGXGG | 50 | 5’-P, 5mC |
| 500 nt-s-9-5mC | GGXGGGACCACCCTTATAAGGCTXGGAGGCXGXGAGGCCTTXGCTGGAGT | 50 | 5’-P, 5mC |
| 500 nt-s-10-5mC | TTXGCAGCXGCAGTCTTXGCCACCAGTGAGTAXGXGXGGCCXGXGTCCCC | 50 | 5’-P, 5mC |
| Donor-s-1 | GAGCTCTGAGCCCCATCCCCGGGGACGCGGGCCGCGCGTA | 40 | - |
| Donor-s-2 | CTCACTGGTGGCGAAGACTGCGGCAGCGAAACTCCAGCGA | 40 | 5’-P |
| Donor-s-3 | AGGCCTCGCGGCCTCCGAGCCTTATAAGGGTGGTCCCGCC | 40 | 5’-P |
| Donor-s-4 | CCGCTCCGCCCCAGTGCTGAGTCACGGCGCCGGCCGCTCT | 40 | 5’-P |
| Donor-s-5 | TCTGGAGGGTCCCGCGGACTCCCGCCGGCCCCAGCCCCGG | 40 | 5’-P |
| Donor-s-6 | CGGCCGCTGCACCCCGGGCGTCGGCCGCAGAGGGGCGCCC | 40 | 5’-P |
| Donor-s-7 | TGGAGTCCCCGGAGTCGCCGCGCAGCTGGCCGGGGAAGCC | 40 | 5’-P |
| Donor-s-8 | TTTCCCTCTTTCCCAGGTCCCCAGCGGGGCCTAGGGAGTA | 40 | 5’-P |
| Donor-s-9 | AACAGACAGCAGGAAGAGGACCGCAGCGAAGTGTGCGCAG | 40 | 5’-P |
| Donor-s-10 | CGAACTGGCGCGCCGGGACATCGCAGAGGGAAACTTGTTT | 40 | 5’-P |
| Donor-s-11 | ATTGCAGCTTATAATGGTTACAAATAAAGCAATAGCATCA | 40 | 5’-P |
| Donor-s-12 | CAAATTTCACAAATAAAGCATTTTTTTCACTGCATTCTAG | 40 | 5’-P |
| Donor-s-13 | TTGTGGTTTGTCCAAACTCATCAATGTATCTTACTAGGTG | 40 | 5’-P |
| Donor-s-14 | CTGTGTCTGGCCTCGGCTCTCTCGTACTGCTCCACGATGG | 40 | 5’-P |
| Donor-s-15 | TGTAGTCCTCGTTGTGGCTGGTGATGTCCAGCTTGATGTC | 40 | 5’-P |
| Donor-c-14 | CAGCCACAACGAGGACTACACCATCGTGGAGCAGTACGAG | 40 | - |
| Donor-c-13 | AGAGCCGAGGCCAGACACAGCACCTAGTAAGATACATTGA | 40 | - |
| Donor-c-12 | TGAGTTTGGACAAACCACAACTAGAATGCAGTGAAAAAAA | 40 | - |
| Donor-c-11 | TGCTTTATTTGTGAAATTTGTGATGCTATTGCTTTATTTG | 40 | - |
| Donor-c-10 | TAACCATTATAAGCTGCAATAAACAAGTTTCCCTCTGCGA | 40 | - |
| Donor-c-9 | TGTCCCGGCGCGCCAGTTCGCTGCGCACACTTCGCTGCGG | 40 | - |
| Donor-c-8 | TCCTCTTCCTGCTGTCTGTTTACTCCCTAGGCCCCGCTGG | 40 | - |
| Donor-c-7 | GGACCTGGGAAAGAGGGAAAGGCTTCCCCGGCCAGCTGCG | 40 | - |
| Donor-c-6 | CGGCGACTCCGGGGACTCCAGGGCGCCCCTCTGCGGCCGA | 40 | - |
| Donor-c-5 | CGCCCGGGGTGCAGCGGCCGCCGGGGCTGGGGCCGGCGGG | 40 | - |
| Donor-c-4 | AGTCCGCGGGACCCTCCAGAAGAGCGGCCGGCGCCGTGAC | 40 | - |
| Donor-c-3 | TCAGCACTGGGGCGGAGCGGGGCGGGACCACCCTTATAAG | 40 | - |
| Donor-c-2 | GCTCGGAGGCCGCGAGGCCTTCGCTGGAGTTTCGCTGCCG | 40 | - |
| Donor-c-1 | CAGTCTTCGCCACCAGTGAGTACGCGCGGCCCGCGTCCCC | 40 | - |
| Donor-s-2-5mC | CTCACTGGTGGXGAAGACTGXGGCAGXGAAACTCCAGXGA | 40 | 5’-P, 5mC |
| Donor-s-3-5mC | AGGCCTXGXGGCCTCXGAGCCTTATAAGGGTGGTCCXGCC | 40 | 5’-P, 5mC |
| Donor-s-4-5mC | CXGCTCXGCCCCAGTGCTGAGTCAXGGXGCXGGCXGCTCT | 40 | 5’-P, 5mC |
| Donor-s-5-5mC | TCTGGAGGGTCCXGXGGACTCCXGCXGGCCCCAGCCCXGG | 40 | 5’-P, 5mC |
| Donor-s-2-1/2-5mC | CTCACTGGTGGCGAAGACTGCGGCAGCGAAACTCCAGCGA | 40 | 5’-P |
| Donor-s-3-1/2-5mC | AGGCCTXGCGGCCTCXGAGCCTTATAAGGGTGGTCCCGCC | 40 | 5’-P |
| Donor-s-4-1/2-5mC | CXGCTCCGCCCCAGTGCTGAGTCAXGGCGCXGGCCGCTCT | 40 | 5’-P |
| Donor-s-5-1/2-5mC | TCTGGAGGGTCCXGCGGACTCCXGCCGGCCCCAGCCCXGG | 40 | 5’-P |
| Donor-s-2-1/3-5mC | CTCACTGGTGGXGAAGACTGCGGCAGCGAAACTCCAGXGA | 40 | 5’-P |
| Donor-s-3-1/3-5mC | AGGCCTCGCGGCCTCXGAGCCTTATAAGGGTGGTCCCGCC | 40 | 5’-P |
| Donor-s-4-1/3-5mC | CCGCTCXGCCCCAGTGCTGAGTCACGGCGCXGGCCGCTCT | 40 | 5’-P |
| Donor-s-5-1/3-5mC | TCTGGAGGGTCCCGXGGACTCCCGCCGGCCCCAGCCCXGG | 40 | 5’-P |
| Donor-s-2-2/3-5mC | CTCACTGGTGGCGAAGACTGXGGCAGXGAAACTCCAGCGA | 40 | 5’-P |
| Donor-s-3-2/3-5mC | AGGCCTXGXGGCCTCCGAGCCTTATAAGGGTGGTCCXGCC | 40 | 5’-P |
| Donor-s-4-2/3-5mC | CXGCTCCGCCCCAGTGCTGAGTCAXGGXGCCGGCXGCTCT | 40 | 5’-P |
| Donor-s-5-2/3-5mC | TCTGGAGGGTCCXGCGGACTCCXGCXGGCCCCAGCCCCGG | 40 | 5’-P |

**Table S2.** s-DNA and c-DNA component of long ssDNA

| **Name** | **Figure** | **s-DNA** | **c-DNA** |
| --- | --- | --- | --- |
| 100 nt ssDNA | S2, 3, 4 | 100nt-s-1 to 100nt-s-5 | 100nt-c-1 to 100nt-c-4 |
| 200 nt ssDNA | S5 | 200nt-s-1 to 200nt-s-10 | 200nt-c-1 to 200nt-c-9 |
| 120 nt ssDNA | S5 | 200nt-s-1-cy3, 200nt-s-2 to 200nt-s-6 | 200nt-c-1 to 200nt-c-5 |
| 140 nt ssDNA | S5 | 200nt-s-1-cy3, 200nt-s-2 to 200nt-s-7 | 200nt-c-1 to 200nt-c-6 |
| 160 nt ssDNA | S5 | 200nt-s-1-cy3, 200nt-s-2 to 200nt-s-8 | 200nt-c-1 to 200nt-c-7 |
| 180 nt ssDNA | S5 | 200nt-s-1-cy3, 200nt-s-2 to 200nt-s-9 | 200nt-c-1 to 200nt-c-8 |
| 200 nt ssDNA | S5 | 200nt-s-1-cy3, 200nt-s-2 to 200nt-s-10 | 200nt-c-1 to 200nt-c-9 |
| 80 nt ssDNA | 2a | 720 nt-1-s-1-cy3, 720 nt-1-s-2 | 720 nt-1-c-1 |
| 120 nt ssDNA | 2a | 720 nt-1-s-1-cy3, 720 nt-1-s-2 to 720 nt-1-s-3 | 720 nt-1-c-1 to 720 nt-1-c-2 |
| 160 nt ssDNA | 2a | 720 nt-1-s-1-cy3, 720 nt-1-s-2 to 720 nt-1-s-4 | 720 nt-1-c-1 to 720 nt-1-c-3 |
| 200 nt ssDNA | 2a | 720 nt-1-s-1-cy3, 720 nt-1-s-2 to 720 nt-1-s-5 | 720 nt-1-c-1 to 720 nt-1-c-4 |
| 240 nt ssDNA | 2a | 720 nt-1-s-1-cy3, 720 nt-1-s-2 to 720 nt-1-s-6 | 720 nt-1-c-1 to 720 nt-1-c-5 |
| 280 nt ssDNA | 2a | 720 nt-1-s-1-cy3, 720 nt-1-s-2 to 720 nt-1-s-7 | 720 nt-1-c-1 to 720 nt-1-c-6 |
| 320 nt ssDNA | 2a | 720 nt-1-s-1-cy3, 720 nt-1-s-2 to 720 nt-1-s-8 | 720 nt-1-c-1 to 720 nt-1-c-7 |
| 360 nt ssDNA | 2a | 720 nt-1-s-1-cy3, 720 nt-1-s-2 to 720 nt-1-s-9 | 720 nt-1-c-1 to 720 nt-1-c-8 |
| 720 nt ssDNA | 2b, left |  |  |
| 360nt-1 | 2b, right | 720 nt-1-s-10 to 720 nt-1-s-18 | 720 nt-1-c-10 to 720 nt-1-c-17 |
| 360nt-2 | 2b, right | 720 nt-2-s-1 to 720 nt-2-s-9 | 720 nt-1-c-1 to 720 nt-1-c-8 |
| 614 nt-1 | 2b, right | 614 nt-1-s-1 to 614 nt-1-s-15 | 614 nt-1-c-1 to 614 nt-1-c-14 |
| 614 nt-2 | 2b, right | 614 nt-2-s-1 to 614 nt-2-s-15 | 614 nt-2-c-1 to 614 nt-2-c-14 |
| 720 nt ssDNA | 2b, right | 720 nt-3-s-1 to 720 nt-3-s-18 | 720 nt-3-c-1 to 720 nt-3-c-17 |
| SF | 3b-c | 720 nt-2-s-1 to 720 nt-2-s-5, 720 nt-2-s-6-FAM, 720 nt-2-s-7 to 720 nt-2-s-9 | 720 nt-2-c-1 to 720 nt-2-c-8 |
| SC | 3b-c | 720 nt-2-s-1 to 720 nt-2-s-6, 720 nt-2-s-7-cy5, 720 nt-2-s-8 to 720 nt-2-s-9 | 720 nt-2-c-1 to 720 nt-2-c-8 |
| DM | 3b-c | 720 nt-2-s-1 to 720 nt-2-s-5, 720 nt-2-s-6-FAM, 720 nt-2-s-7-cy5, 720 nt-2-s-8 to 720 nt-2-s-9 | 720 nt-2-c-1 to 720 nt-2-c-8 |
| N-DNA | S9 | 720 nt-1-s-1 to 720 nt-1-s-9 | 720 nt-1-c-1 to 720 nt-1-c-8 |
| 5’-pt-DNA | S9 | 720 nt-1-s-1-pt, 720 nt-1-s-2 to 720 nt-1-s-9 | 720 nt-1-c-1 to 720 nt-1-c-8 |
| 3’-pt-DNA | S9 | 720 nt-1-s-1 to 720 nt-1-s-8, 720 nt-1-s-9-pt | 720 nt-1-c-1 to 720 nt-1-c-8 |
| 5’, 3’-pt-DNA | S9 | 720 nt-1-s-1-pt, 720 nt-1-s-2 to 720 nt-1-s-8, 720 nt-1-s-9-pt | 720 nt-1-c-1 to 720 nt-1-c-8 |
| i-pt-DNA | S9 | 720 nt-1-s-1 to 720 nt-1-s-2, 720 nt-1-s-3-pt, 720 nt-1-s-4 to 720 nt-1-s-9 | 720 nt-1-c-1 to 720 nt-1-c-8 |
| 500 nt-NO-5mC | 3e | 500 nt-s-1 to 500 nt-s-10 | 500 nt-c-1 to 500-c-9 |
| 500 nt M-DNA-1 | 3e | 500 nt-s-1 to 500 nt-s-6, 500 nt-s-7-5mC to 500 nt-s-8-5mC, 500 nt-s-9 to 500 nt-s-10 | 500 nt-c-1 to 500-c-9 |
| 500 nt M-DNA-2 | 3e | 500 nt-s-1 to 500 nt-s-8, 500 nt-s-9-5mC, 500 nt-s-10 | 500 nt-c-1 to 500-c-9 |
| N-5mC | S11-12 | Donor-s-1 to Donor-s-15 | Donor-c-1 to Donor-c-14 |
| F-5mC | S11-12 | Donor-s-1, Donor-s-2-5mC to Donor-s-5-5mC, Donor-s-6 to Donor-s-15 | Donor-c-1 to Donor-c-14 |
| 1/2-5mC | S11-12 | Donor-s-1, Donor-s-2-1/2-5mC to Donor-s-5-1/2-5mC, Donor-s-6 to Donor-s-15 | Donor-c-1 to Donor-c-14 |
| 1/3-5mC | S11-12 | Donor-s-1, Donor-s-2-1/3-5mC to Donor-s-5-1/3-5mC, Donor-s-6 to Donor-s-15 | Donor-c-1 to Donor-c-14 |
| 2/3-5mC | 1/3-5mC | Donor-s-1, Donor-s-2-2/3-5mC to Donor-s-5-2/3-5mC, Donor-s-6 to Donor-s-15 | Donor-c-1 to Donor-c-14 |

**Table S3.** Sequences of synthesized ssDNA donors for methylation manipulation

| **Name** | **Figure** |
| --- | --- |
| N-5mC | GAGCTCTGAGCCCCATCCCCGGGGACGCGGGCCGCGCGTACTCACTGGTGGCGAAGACTGCGGCAGCGAAACTCCAGCGAAGGCCTCGCGGCCTCCGAGCCTTATAAGGGTGGTCCCGCCCCGCTCCGCCCCAGTGCTGAGTCACGGCGCCGGCCGCTCTTCTGGAGGGTCCCGCGGACTCCCGCCGGCCCCAGCCCCGGCGGCCGCTGCACCCCGGGCGTCGGCCGCAGAGGGGCGCCCTGGAGTCCCCGGAGTCGCCGCGCAGCTGGCCGGGGAAGCCTTTCCCTCTTTCCCAGGTCCCCAGCGGGGCCTAGGGAGTAAACAGACAGCAGGAAGAGGACCGCAGCGAAGTGTGCGCAGCGAACTGGCGCGCCGGGACATCGCAGAGGGAAACTTGTTTATTGCAGCTTATAATGGTTACAAATAAAGCAATAGCATCACAAATTTCACAAATAAAGCATTTTTTTCACTGCATTCTAGTTGTGGTTTGTCCAAACTCATCAATGTATCTTACTAGGTGCTGTGTCTGGCCTCGGCTCTCTCGTACTGCTCCACGATGGTGTAGTCCTCGTTGTGGCTGGTGATGTCCAGCTTGATGTC |
| 1/3-5mC | GAGCTCTGAGCCCCATCCCCGGGGACGCGGGCCGCGCGTACTCACTGGTGG/5mC/GAAGACTGCGGCAGCGAAACTCCAG/5mC/GAAGGCCTCGCGGCCTC/5mC/GAGCCTTATAAGGGTGGTCCCGCCCCGCTC/5mC/GCCCCAGTGCTGAGTCACGGCGC/5mC/GGCCGCTCTTCTGGAGGGTCCCG/5mC/GGACTCCCGCCGGCCCCAGCCC/5mC/GGCGGCCGCTGCACCCCGGGCGTCGGCCGCAGAGGGGCGCCCTGGAGTCCCCGGAGTCGCCGCGCAGCTGGCCGGGGAAGCCTTTCCCTCTTTCCCAGGTCCCCAGCGGGGCCTAGGGAGTAAACAGACAGCAGGAAGAGGACCGCAGCGAAGTGTGCGCAGCGAACTGGCGCGCCGGGACATCGCAGAGGGAAACTTGTTTATTGCAGCTTATAATGGTTACAAATAAAGCAATAGCATCACAAATTTCACAAATAAAGCATTTTTTTCACTGCATTCTAGTTGTGGTTTGTCCAAACTCATCAATGTATCTTACTAGGTGCTGTGTCTGGCCTCGGCTCTCTCGTACTGCTCCACGATGGTGTAGTCCTCGTTGTGGCTGGTGATGTCCAGCTTGATGTC |
| 1/2-5mC | GAGCTCTGAGCCCCATCCCCGGGGACGCGGGCCGCGCGTACTCACTGGTGG/5mC/GAAGACTGCGGCAG/5mC/GAAACTCCAGCGAAGGCCT/5mC/GCGGCCTC/5mC/GAGCCTTATAAGGGTGGTCCCGCCC/5mC/GCTCCGCCCCAGTGCTGAGTCA/5mC/GGCGC/5mC/GGCCGCTCTTCTGGAGGGTCC/5mC/GCGGACTCC/5mC/GCCGGCCCCAGCCC/5mC/GGCGGCCGCTGCACCCCGGGCGTCGGCCGCAGAGGGGCGCCCTGGAGTCCCCGGAGTCGCCGCGCAGCTGGCCGGGGAAGCCTTTCCCTCTTTCCCAGGTCCCCAGCGGGGCCTAGGGAGTAAACAGACAGCAGGAAGAGGACCGCAGCGAAGTGTGCGCAGCGAACTGGCGCGCCGGGACATCGCAGAGGGAAACTTGTTTATTGCAGCTTATAATGGTTACAAATAAAGCAATAGCATCACAAATTTCACAAATAAAGCATTTTTTTCACTGCATTCTAGTTGTGGTTTGTCCAAACTCATCAATGTATCTTACTAGGTGCTGTGTCTGGCCTCGGCTCTCTCGTACTGCTCCACGATGGTGTAGTCCTCGTTGTGGCTGGTGATGTCCAGCTTGATGTC |
| 2/3-5mC | GAGCTCTGAGCCCCATCCCCGGGGACGCGGGCCGCGCGTACTCACTGGTGGCGAAGACTG/5mC/GGCAG/5mC/GAAACTCCAGCGAAGGCCT/5mC/G/5mC/GGCCTCCGAGCCTTATAAGGGTGGTCC/5mC/GCCC/5mC/GCTCCGCCCCAGTGCTGAGTCA/5mC/GG/5mC/GCCGGC/5mC/GCTCTTCTGGAGGGTCC/5mC/GCGGACTCC/5mC/GC/5mC/GGCCCCAGCCCCGGCGGCCGCTGCACCCCGGGCGTCGGCCGCAGAGGGGCGCCCTGGAGTCCCCGGAGTCGCCGCGCAGCTGGCCGGGGAAGCCTTTCCCTCTTTCCCAGGTCCCCAGCGGGGCCTAGGGAGTAAACAGACAGCAGGAAGAGGACCGCAGCGAAGTGTGCGCAGCGAACTGGCGCGCCGGGACATCGCAGAGGGAAACTTGTTTATTGCAGCTTATAATGGTTACAAATAAAGCAATAGCATCACAAATTTCACAAATAAAGCATTTTTTTCACTGCATTCTAGTTGTGGTTTGTCCAAACTCATCAATGTATCTTACTAGGTGCTGTGTCTGGCCTCGGCTCTCTCGTACTGCTCCACGATGGTGTAGTCCTCGTTGTGGCTGGTGATGTCCAGCTTGATGTC |
| F-5mC | AGGGAGTTGATTTCAGACTATGCACCAGATTGTTTTGTGTTAATAGAAATCTCACTGGTGG/5mC/GAAGACTG/5mC/GGCAG/5mC/GAAACTCCAG/5mC/GAAGGCCT/5mC/G/5mC/GGCCTC/5mC/GAGCCTTATAAGGGTGGTCC/5mC/GCCC/5mC/GCTC/5mC/GCCCCAGTGCTGAGTCA/5mC/GG/5mC/GC/5mC/GGC/5mC/GCTCTTCTGGAGGGTCC/5mC/G/5mC/GGACTCC/5mC/GC/5mC/GGCCCCAGCCC/5mC/GGCGGCCGCTGCACCCCGGGCGTCGGCCGCAGAGGGGCGCCCTGGAGTCCCCGGAGTCGCCGCGCAGCTGGCCGGGGAAGCCTTTCCCTCTTTCCCAGGTCCCCAGCGGGGCCTAGGGAGTAAACAGACAGCAGGAAGAGGACCGCAGCGAAGTGTGCGCAGCGAACTGGCGCGCCGGGACATCGCAGAGGGAAACTTGTTTATTGCAGCTTATAATGGTTACAAATAAAGCAATAGCATCACAAATTTCACAAATAAAGCATTTTTTTCACTGCATTCTAGTTGTGGTTTGTCCAAACTCATCAATGTATCTTACTAGGTGCTGTGTCTGGCCTCGGCTCTCTCGTACTGCTCCACGATGGTGTAGTCCTCGTTGTGGCTGGTGATGTCCAGCTTGATGTC |

**Table S4.** Sequences of synthesized ssDNA with high G/C ratio, high A/T ratio and repetitive sequences

| **Name** | **Sequence** |
| --- | --- |
| High G/C ratio | CCGGCCAGCCGCGCGGCGACTCCGGGGACTCCGGGGCGCCCCTCTGCGGCCGACGCCCGGGGTGCAGCGGCCGCCGGGGCTGGGGCCGGCGGGCGTCCGCGGGACCCTCCAGAAGAGCGGCCGGCGCCGCGACTCAGCACTGGGGCGGAGCGGGGCGGGACCACCCTTATGGGGCTCGGAGGCCGCGAGGCCTTCGCTGGAGTTTCGCTGCCGCAGTCTTCGCAGCCGAGCCGCTCCGCCGCCGCACCGCCGCCGCCAGCCTGTCCGCCGCCCGCCCGCCCGCCCGCCGCGTTATAATTG |
| High A/T ratio | AATTAACTAATATATTATCAGAATTTTCAGAATTTTATAAAAGAGTATTAATCATAAATTTTGTACTATTAATAATTTTAGTTTTAATTATTTATGAATATTTCAAAGAACTCCTCTATTAATTATAATATCAGACTACAGTTTTATTCTATTTTATTTCAACAAAGGCGTTTTAGATTCTTAATATCTTAAGGATAGTTCTGGGATAGTAATACTGAGGATACTAATCTAATAGAATAATAATACAATAATAATAACTAAGTGAATAATAAATAAATAAATAAATAATATGGCGCCGGT |
| Repetitive sequence | GAGCTCTGAGCCCCATCCCCGGGGACGCGGGCCGCGCGTACTCACTGGCTGTTCACCGGGCAACGTAGGTCGCTGTTCACCGGGCAACGTAGGTCGCTGTTCACCGGGCAACGTAGGTCGCTGTTCACCGGGCAACGTAGGTCGCTGTTCACCGGGCAACGTAGGTCGCTGTTCACCGGGCAACGTAGGTCGCTGTTCACCGGGCAACGTAGGTCGCTGTTCACCGGGCAACGTAGGTCG |

*Repeating unit is highlighted

**Figure S1.** Assembly results of s-DNA and c-DNA with different melting temperature. Image shown is the native PAGE (1×TBE) result of the assembly of s-DNA and c-DNA with melting temperature at 25 °C, 35 °C and 45 °C, respectively. Adjacent lanes represent two groups of replicates performed at the same melting temperature.

**Figure S2.** One-pot synthesis of 100 nt and 200 nt ssDNA. The 100 nt ssDNA is synthesized from 5 s-DNAs and 4 c-DNAs, while the 200 nt ssDNA is synthesized from 10 s-DNAs and 9 c-DNAs. Gel electrophoresis results show synthesis efficiencies of 98% and 84% for 100 nt and 200 nt products, respectively. Efficiency was calculated by dividing the band intensity of the topmost band (corresponding to the target ssDNA) by the sum of all bands except the lowest band, which contains residual c-DNAs.

**Figure S3.** Characterization of concentration insensitivity of synthesizing 100 nt ssDNA. The concentration of s-DNA and s-DNA in the synthetic system were diluted to 10 µM, 5 µM, 2.5 µM, 1.25 µM, 600 nM and 200 nM at ligation procedure. The total amount of the mixture was adjusted to be equivalent during gel electrophoresis. The ssDNA is synthesized from 5 s-DNAs and 4 c-DNAs.

**Figure S4.** Tolerance of stochiometric ratio of s-DNA and c-DNA during synthesis of 100 nt ssDNA. (a) Scheme of procedure of characterization of the stochiometric ratio tolerance of synthesis of 100 nt ssDNA. **b-c,** 10% native PAGE. (b) and 10% denaturing PAGE. (c) analysis of assembly of the 9 ssDNA and ligated product, respectively. Lanes from left to right: equivalent amount of 9 ssDNA, addition of 50% excessive amount of T-1, T-2, T-3, C-4 and C-3 and 10%, 20%, 50% and 100% excessive amount of T-3. The band corresponding to full-length assembly and targeted ssDNA are dominant and highlighted in box.

**Figure S5.** One-pot synthesis of 140 to 200 nt ssDNA using 20 nt s-DNA. Left, 8% denaturing PAGE (7 mol/L urea) analysis of synthesized 120 nt, 140 nt, 160 nt, 180 nt and 200 nt ssDNA. s-DNA at the 5ʹ site in the synthesis system was modified with a 5ʹ FAM fluorescent group. The gel was visualized under FAM channel. Right, calculation of synthesis efficiency based on band intensity analysis. 120 nt, 140 nt, 160 nt, 180 nt and 200 nt ssDNA were synthesized from 6, 7, 8, 9, 10 s-DNAs, respectively.

**Figure S6.** Characterization of the synthesis efficiency under different reaction time. Left, 6% denaturing PAGE (7 mol/L urea) analysis of synthesis of 360 nt-long ssDNA Right, calculation of synthesis efficiency at different reaction time based on band intensity analysis. ssDNAs are synthesized from 9 s-DNAs and 8 c-DNAs.

**Figure S7.** Synthesis of ssDNA with different lengths. (a) Schematic illustration of the purification procedure for target ssDNA from synthesis mixtures. Target bands were excised from denaturing PAGE gels, c rushed, and soaked in nuclease-free water overnight. The supernatant containing released DNA was collected and purified by desalting via molecular cut-off centrifugal filter unit. (b) Demonstration of one-pot synthesis of ultra-long 1228 nt ssDNA. 6% denaturing PAGE analysis showing: lane 1, synthesis mixture of 1228 nt ssDNA; lane 2, 200 nt ssDNA ladder; lane 3, 720 nt ssDNA reference; lane 4, purified 1228 nt ssDNA; lane 5, 600 nt ssDNA reference; lane 6, 100 nt ssDNA marker. The efficiency of synthesizing 1228 nt ssDNA is hard to estimate because the bands between target ssDNA and incompletely ligated intermediates are hard to discriminate.

**Figure S8. Synthesis of long ssDNA with extreme sequence content.** (a) 8% denaturing PAGE analysis of synthesis mixture and purified products of 300 nt G/C-rich (left) and A/T-rich (right) ssDNAs. (b) Schematic illustration of synthesis strategy for 192 nt ssDNA containing eight 24 nt tandem repeats. (c) 8% denaturing PAGE analysis of synthesis mixture and purified product of 192 nt ssDNA with repetitive sequences.

**Figure S9. Heatmap of error rate of different ssDNA: 720 nt (a), 300 nt (b), 360 nt (c) and 1228 nt (d) ssDNA.** The sequencing heatmap was counted from Sanger sequencing results of 40 clones for 720 nt ssDNA and 20 clones for 300 nt, 360 nt and 1228 nt ssDNAs.. Each of the 18×40, 50×6, 9×40 and 20×60+1×28 grid represents the sum of sequencing results of single base and the non-complementary read between the forward and reverse strands. The error rate scale is shown on the right (0-1).

**Figure S10.** Synthesis of 360 nt ssDNA with site-specifically phosphorothioate (pt) modifications. ssDNAs are synthesized from 9 s-DNAs and 8 c-DNAs. (a) Schematic of synthesis and characterization of 360 nt ssDNA with distal phosphorothioate (pt) modification. Modifications on different sites were characterized by RecJ_f_ (digests from 5ʹ to 3ʹ) and ExoI (digests from 3ʹ to 5ʹ) exonuclease digestion assay. (b) 6% denaturing PAGE analysis of synthesized ssDNA with or without pt modification. Four kinds of ssDNA were synthesized: N-DNA (without pt modification), 5ʹ-ps-DNA (3 consecutive pt modifications at 5ʹ), 3ʹ-ps-DNA (3 consecutive pt modifications at 3ʹ) and 5ʹ,3ʹ-ps-DNA (3 consecutive modifications at both 5ʹ and 3ʹ site). From left to right, each 3 lanes of each ssDNA correspond to the product without digestion, RecJ_f_ digestion and ExoI digestion. **c,** Schematic of synthesis and characterization of 360 nt ssDNA with internal pt modifications, which is the cleavage site of AscI endonuclease. Synthesized ssDNA was hybridized with 40 nt ssDNA to generate the cleavable dsDNA template for AscI. The precise modification was characterized by protection from AscI cleavage of synthesized ssDNA. **d,** 6% denaturing PAGE analysis of internal pt modification of synthesized 360 nt ssDNA. i-ps-DNA represents ssDNA with pt modification on the AscI cleavage site. Two lanes of correspond to ssDNA without cleavage and with AscI cleavage after hybridization.

**Figure S11.** Characterization of ssDNA with site-specific 5mC modifications. (a) 6% denaturing PAGE characterization of 500 nt ssDNA with 5mC modifications at different sites. Lanes in image: M, 100 nt ssDNA ladder; 1, ssDNA without 5mC modifications; 2, ssDNA with 5mC modifications at 22^th^ to 36^th^ CpG sites (M-DNA-1); 3, ssDNA with 5mC modifications at 15^th^ to 49^th^ CpG sites (M-DNA-2). The dashed line represents non-adjacent bands within the same gel. ssDNAs are synthesized from 10 s-DNAs and 9 c-DNAs. (b) 5mC modification rate statistical graphs for each CpG sites (from 15^th^ to 49^th^ CpG sites in ssDNA, here denoted as 1^st^ to 35^th^) based on the bisulfite sequencing results.

**Figure S12.** Synthesis of ssDNA donor with different 5mC modifications. 6% denaturing PAGE characterization of synthesized ssDNA. Lanes in gel: M, 200 nt ssDNA ladder; 1, N-5mC; 2, 1/3-5mC; 3, 1/2-5mC; 4, 2/3-5mC; 5, F-5mC. ssDNAs are synthesized from 15 s-DNAs and 14 c-DNAs.

**Figure S13.** Bisulfite sequencing of synthesized ssDNA donor. The images displayed the statistical results of 8 clones after bisulfite treatment, PCR amplification and cloning into vector. Only the CpG sites which potentially exhibit methylation were shown.

**Figure S14.** Scheme of construction of cell line and flow cytometry and Bisulfite-PCR characterization. (a) Scheme of construction of HEK 293T cell line using CRISPR-Cas9-directed HDR. The SV40 promotor (denoted as SV40 P) and sfCherry2_1-10_ split fluorescent protein were inserted upstream of GSTP1 promotor (denoted as GSTP1 P) The eGFP fluorescent protein sequence was inserted at the N-terminus of GSTP1, which can serve as the selection signal during the construction of the cell line. (b) Flow cytometry analysis of representative single-cell cloning. After electroporation, fluorescence-activated cell sorting (FACS) was adopted to separate eGFP^+^ cells. Single-cell cloning of sorted cells were cultured and analyzed by flow cytometry, among which homogeneous eGFP^+^ cells were selected for methylated ssDNA donor integration. (c) Bisulfite-PCR analysis of 5mC ratio near GSTP1 promotor of sorted cells. The results indicated that the integration of exogeneous elements into genome of HEK 293T cells didn’t influence the hypomethylation state near GSTP1 promotor.

**Figure S15.** Characterization of the integration of ssDNA donor. (a) flow cytometry analysis of cells after integration of different ssDNA donor. Shown is the gating of sfCheery2-positive cells. (b) The genomic coordination of cellular genome after ssDNA donor integration. (c) Genotyping PCR analysis of cells after ssDNA donor integration. Upper image, specific primers were designed to amplify regions that only exist in the genome of cells where the integration of target ssDNA donor has occurred. Lower image, 2% agarose gel analysis of the results of PCR. The generation of targeted 219 bp amplicon demonstrated the successful integration of the ssDNA donor.

**Figure S16.** Characterization of 5mC ratio of each CpG dyad near GSTP1 promotor. (a) scheme of method of characterization of 5mC ratio using bisulfite-PCR with specific primers. After electrophoresis, genomic DNA of cells was extracted and treated with sodium bisulfite. Specific primers were then used to amplify targeted regions in the genome of cells with successful ssDNA donor integration. Amplicons were purified by gel extraction, ligated with adaptors and sequenced by high-throughput sequencing. (b) 1.5% agarose gel electrophoresis analysis of product of bisulfite-PCR. The amplification of bisulfite-treated N-5mC and genomic DNA of cells without electroporation was tested as negative control. Targeted amplicons were marked with red circle. Extra bands visible outside the boxed target regions represent non-specific amplification products commonly observed in bisulfite-PCR due to sequence heterogeneity introduced by bisulfite conversion. Only the boxed bands corresponding to the expected product size were used for subsequent sequencing analysis.

**References**

[1]	S. Chen, Y. Zhou, Y. Chen, J. Gu, “fastp: an ultra-fast all-in-one FASTQ preprocessor” *Bioinformatics* **2018**, *34*, i884–i890.

[2]	F. Krueger, S. R. Andrews, “Bismark: a flexible aligner and methylation caller for Bisulfite-Seq applications” *Bioinformatics* **2011**, *27*, 1571–1572.
